# Copper Phosphinate Complexes as Molecular Precursors for Ethanol Dehydrogenation Catalysts

**DOI:** 10.1021/acs.inorgchem.3c01678

**Published:** 2023-11-30

**Authors:** Tomas Pokorny, Iaroslav Doroshenko, Petr Machac, Lucie Simonikova, Miroslava Bittova, Zdenek Moravec, Katerina Karaskova, David Skoda, Jiri Pinkas, Ales Styskalik

**Affiliations:** † Department of Chemistry, Faculty of Science, Masaryk University, Kotlarska 2, CZ-61137 Brno, Czech Republic; ‡ Institute of Environmental Technology,CEET, VSB-TUO, CZ-70800 Ostrava, Czech Republic; § Centre of Polymer Systems, Tomas Bata University in Zlin, Tr. Tomase Bati 5678, CZ-76001 Zlin, Czech Republic

## Abstract

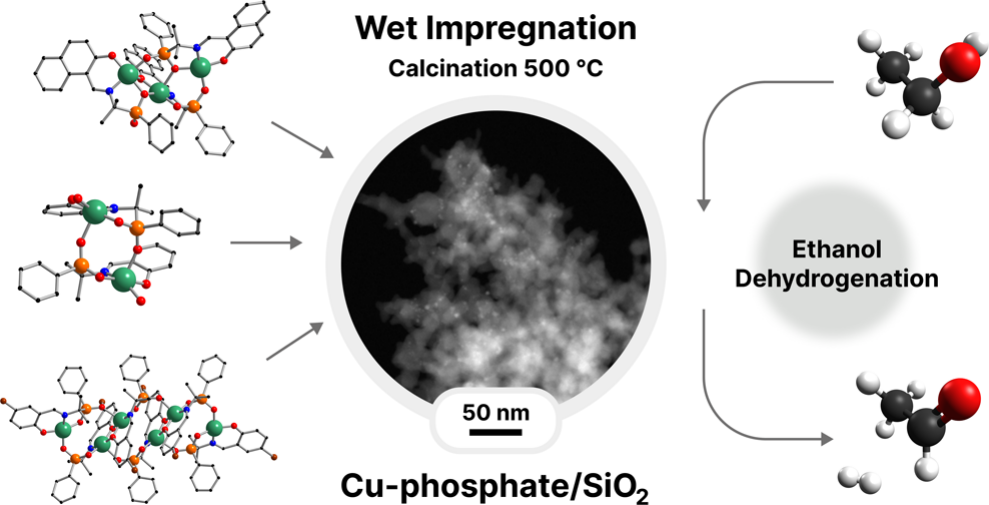

Nowadays, the production of acetaldehyde heavily relies on the petroleum industry. Developing new catalysts for the ethanol dehydrogenation process that could sustainably substitute current acetaldehyde production methods is highly desired. Among the ethanol dehydrogenation catalysts, copper-based materials have been intensively studied. Unfortunately, the Cu-based catalysts suffer from sintering and coking, which lead to rapid deactivation with time-on-stream. Phosphorus doping has been demonstrated to diminish coking in methanol dehydrogenation, fluid catalytic cracking, and ethanol-to-olefin reactions. This work reports a pioneering application of the well-characterized copper phosphinate complexes as molecular precursors for copper-based ethanol dehydrogenation catalysts enriched with phosphate groups (**Cu-phosphate/SiO**_**2**_). Three new catalysts (**CuP-1**, **CuP-2**, and **CuP-3**), prepared by the deposition of complexes {Cu(SAAP)}_*n*_ (**1**), [Cu_6_(BSAAP)_6_] (**2**), and [Cu_3_(NAAP)_3_] (**3**) on the surface of commercial SiO_2_, calcination at 500 °C, and reduction in the stream of the forming gas 5% H_2_/N_2_ at 400 °C, exhibited unusual properties. First, the catalysts showed a rapid increase in catalytic activity. After reaching the maximum conversion, the catalyst started to deactivate. The unusual behavior could be explained by the presence of the phosphate phase, which made Cu^2+^ reduction more difficult. The phosphorus content gradually decreased during time-on-stream, copper was reduced, and the activity increased. The deactivation of the catalyst could be related to the copper diffusion processes. The most active **CuP-1** catalyst reaches a maximum of 73% ethanol conversion and over 98% acetaldehyde selectivity at 325 °C and WHSV = 2.37 h^–1^.

## Abstract 2

This work introduces an advantageous approach using soluble copper phosphinate complexes as molecular precursors for highly dispersed Cu-phosphate/SiO_2_ catalysts in ethanol dehydrogenation. The resulting catalysts demonstrated an unusual initial increase in activity, attributed to the phosphate phase hindering Cu^2+^ reduction. Despite the gradual deactivation, the catalysts exhibited a notable performance with a max 73% ethanol conversion and over 98% acetaldehyde selectivity at 325 °C and WHSV = 2.37 h^−1^.

## Introduction

Nowadays, the production of chemical compounds tends to focus on ecology and sustainability.^1,2^ Acetaldehyde is a large-scale substance produced worldwide for many applications and as a precursor for further synthesis (acetic acid and ethyl acetate).^3^ Current production of acetaldehyde is mainly based on petroleum chemistry.^4−8^ First, the ethylene is produced by energetically demanding steam cracking, and then the acetaldehyde is made by the Wacker oxidation using homogeneous catalysts containing heavy metals.^9,10^ Nonoxidative dehydrogenation of ethanol to acetaldehyde (the first step of the Lebedev process^11,12^) shows the utilization of bioethanol to produce a variety of sustainable and biobased chemicals.^13,14^

According to the literature reports, copper acts as a highly active and selective ethanol dehydrogenation catalyst.^7^ For example, high selectivity to acetaldehyde (up to 100%) has been reported by Chang et al., where rice husk ash (more than 99% SiO_2_) with copper nanoparticles (1–1.5 nm) were used as catalysts.^15^ However, current copper-based catalysts suffer from deactivation by coking and sintering.^14,16−19^ Coking has been shown to be one of the deactivation processes in Cu/SiO_2_ catalysts prepared by dry impregnation, strong electrostatic adsorption, hydrolytic sol–gel, and solvothermal hot injection.^20^ Similarly, Pampararo et al. showed carbon deposition on Cu/SiO_2_ catalysts prepared by aerosol-assisted sol–gel. The more active the catalysts, the higher the amount of carbonaceous materials deposited during the catalytic reaction.^21^ In addition to catalyst coking, copper sintering presents a severe problem at higher temperatures. For example, Cu/SiO_2_ catalysts prepared by incipient wetness impregnation lost half of their activity during 4 h at 300 °C. The deactivation was caused by Cu sintering.^22^ Similarly, Cu/SiO_2_ samples with 0.5 and 1 wt % of copper started losing catalytic activity at 300 °C due to the particle sintering. Surprisingly, smaller particles prepared by the deposition–precipitation method (23 nm) exhibited better catalytic activity and stability than larger particles prepared by wet impregnation (84 nm).^16^ These results suggest that it is crucial to study the stability of copper-based catalysts, describe the sintering and coking in detail, and develop new catalysts based on the gained knowledge.^14,16−19^

Applying molecular precursors to prepare structure-controlled catalysts with homogeneously dispersed catalytic species on the support is a well-known and widely used method.^23−25^ However, there is a lack of information on applying phosphorus-containing metal complexes (phosphates, phosphonates, and phosphinates) as precursors to nonoxidative ethanol dehydrogenation catalysts. Numerous studies have been conducted on copper phosphates^26−34^ and phosphonates.^35−53^ However, the application of phosphinate ligands for the formation of copper complexes has seen limited exploration, with a modest number of copper complexes based on phosphinate ligands present to date.^54−93^ A small amount of the copper complexes with macrocyclic ligands possessing pendant phosphinate groups are also being studied for potential medical applications.^94−101^ The formation of insoluble polymeric compounds is commonplace in such systems, which is a factor that limits their potential application as molecular precursors. Typical strategies employed for the isolation of molecular species include the utilization of bulky ligands,^34,37^ incorporation of additional ancillary ligands,^40,48,49,52,65,73^ and the exchange of labile ligands within the molecular clusters (cluster expansion).^51,52,80^

The addition of phosphorus into Cu/SiO_2_ catalysts prepared by incipient wetness impregnation and ion-exchange method was studied by Yamamoto et al.^102^ Interestingly, phosphorus-containing catalysts exhibited a significant increase in formaldehyde productivity during methanol dehydrogenation. Incorporating phosphorus in Cu/AlPO catalysts is known to influence catalytic activity and stability.^103^ Synthesizing alumina modified with phosphorus reduces the amount of coke formation in the methanol-to-dimethyl ether dehydration reaction.^104^ Similarly, the addition of phosphorus in the form of phosphate has been reported to lead to a decrease in coke formation in hydrocarbon catalysis and consequently to increased catalytic lifetime.^102^ van der Bij et al.^105^ and Xia et al.^106^ pointed to anticoking properties of phosphorus-doped zeolites.

This work presents the structures of new well-soluble polymer (**1**) and molecular (**2** and **3**) copper phosphinate complexes of various nuclearity, their deposition on porous silica support by wet impregnation followed by thermal decomposition leading to **Cu-phosphate/SiO**_**2**_ materials, and the catalytic properties of these catalysts in ethanol dehydrogenation. The main goal of the application of the copper phosphinate complexes in wet impregnation was to disperse copper and phosphorus homogeneously on the surface of the SiO_2_ matrix. Through the utilization of readily synthesized novel Schiff base phosphinate ligands (SAAP^2–^, BSAAP^2–^, and NAAP^2–^) incorporating additional organic groups featuring donor and ionic functionalities in the process of complex preparation, it becomes possible to produce mainly molecular, highly soluble metallophosphinates. These species possess a predetermined stoichiometric ratio of Cu and P, as well as varying nuclearities that depend on the structure of the ligand. Small and uniform particles containing both Cu and P in equal molar amounts were successfully formed. Interestingly, the materials prepared in such a way exhibited a peculiar catalytic behavior, distinctively different from the benchmark catalysts obtained by the dry impregnation methods with and without the presence of phosphorus. The most active **Cu-phosphate/SiO**_**2**_ catalyst was analyzed ex-situ by ICP-OES, XPS, and STEM analyses at the different stages of the catalyst lifetime (i.e., after calcination, after H_2_ treatment, at the top of catalytic activity, spent catalyst) to gain a deep understanding of the unprecedented catalytic properties.

## Experimental Section

### General Procedures

All reactions were performed using general synthetic techniques; no special conditions were used. Commercially available benzyl carbamate (TCI), dichlorophenylphosphine (Sigma), NaOH, salicylaldehyde (Sigma), 5-bromosalicylaldehyde (Sigma), 2-hydroxy-1-naphtalaldehyde (Sigma), methanol-d_4_ (Sigma), benzene-d_6_ (TCI), propan-2-ol (p.a.), ethanol (p.a.), methanol (p.a.), acetone (p.a.), pentane (p.a.), acetic acid (p.a.), Cu(NO_3_)_2_·2.5H_2_O, and Aerosil 300 (Evonik) were used as received.

**Caution!***Acute toxic (oral, Cat 3) and corrosive (Sub-Cat 1B) dichlorophenylphosphine; corrosive (Cat 1, Sub-Cat 1A) NaOH; Acute toxic (oral, Cat 4) and aquatic hazard (Cat 2) salicylaldehyde; Flammable (Cat 2) and acute toxic (Cat 1, 3) methanol-d4; Flammable (Cat 2), skin and eye irritant (Cat 2), toxic (Cat 1), carcinogenic and mutagenic (Cat 1A, 1B), and aquatic hazard (Cat 3) benzene-d6; Flammable (Cat 2), eye irritant (Cat 2), and specific target organ toxic (Cat 3) propan-2-ol; Flammable (Cat 2) and eye irritant (Cat 2) ethanol; Flammable (Cat 2) and acute toxic (oral, inhalation, and dermal, Cat 3) methanol; Flammable (Cat 2), eye irritant (Cat 2), and specific target organ toxic (Cat 3) acetone; Flammable (Cat 2), specific target organ toxic (Cat 3), aspiration hazardous (Cat 1), and aquatic hazard (Cat 2) pentane; Flammable (Cat 3), corrosive (Sub-Cat 1A), and serious eye damage (Cat 1) acetic acid; Oxidizing (Cat 1), corrosive (Cat 1, Sub-Cat 1B), acute toxic (oral, Cat 4), eye damage (Cat 1), and aquatic hazard (Cat 1) copper nitrate constitute significant safety hazards and must be handled with care and caution.*

### Characterization Methods

Elemental maps, nanoparticle size, and distribution were measured by scanning transmission electron microscopy with energy-dispersive X-ray spectroscopy (STEM-EDS) on a Thermo Fisher Scientific Talos F200 instrument equipped with a Bruker X-flash EDS detector. The device operated at 40–200 kV of accelerating potential. The size of the nanoparticles was determined using an ImageJ image processing program.^107^ Nanoparticle diameters were measured across the widest side. The surface chemical composition was analyzed by X-ray photoelectron spectroscopy (XPS) on a Kratos Axis Supra device equipped with a monochromatic X-ray source with Al K_α_ (*E* = 1486.6 eV) excitation. Binding energy 284.8 eV for C 1s was used for calibration. An Autosorb iQ3 (Quantachrome Instrument) porosimeter was used for measuring the specific surface area by nitrogen adsorption. Both isotherms (adsorption and desorption) were measured at liquid nitrogen temperature (−195.7 °C). Before measurements, samples were degassed at a temperature of 200 °C. BET analysis was used to determine the specific surface area from isotherms measured in the 0.05–0.30 relative pressure range. A Netsch STA 449 Jupiter instrument was used for thermogravimetric (TG) analyses. Samples were heated to 1000 °C in Pt crucibles with a heating rate of 5 °C min^–1^ in a synthetic air atmosphere with a flow of 100 cm^3^ min^–1^. Single-crystal X-ray diffraction measurements were performed on a Rigaku diffraction system (MicroMax007HF DW rotating anode source with multilayer optic, partial χ axis goniometer, Saturn 724+ HG detector, and Cryostream cooling device). The Mo–K_α_ (λ = 0.7107 Å) radiation was used. Data were corrected for Lorentz and polarization effects; absorption was taken into account on a semiempirical basis using multiple-scans.^108−110^*CrystalClear* (Rigaku 2014) and *CrysAlisPro* (Agilent Technologies 2013) software packages were used for data collection and reduction. The structures were solved using the *SHELXT*(111) program and refined (full matrix least-squares refinement on *F*_o_^2^) using the *SHELXL*(112) program. An EMPYREAN instrument from PANalytical was used for powder X-ray diffraction analyses. Samples were placed on a spinning sample bed. The Co K_α_ radiation (λ = 1.78903 Å) was used (20 mA, 30 kV). A semiconductor detector was used in 1D mode. Also, a MiniFlex 600 instrument by Rigaku was used for powder X-ray diffraction analyses. The Co K_α_ radiation (λ = 1.78903 Å) was used (15 mA, 40 kV). Data processing was performed with Rigaku PDXL2 software. Elemental contents were measured by inductively coupled plasma optical emission spectroscopy (ICP-OES). ICP-OES analyses were done on an ICP-OES spectrometer iCAP PRO XPS Duo (Thermo, RF Power 1.10 kW, nebulizer gas flow 0.65 dm^3^ min^–1^, radial viewing high 11.0 mm). Emission lines 177.495, 178.284, and 213.618 nm for P and 324.754 and 327.396 nm for Cu were used. The ESI-MS spectra were measured on an Agilent 6224 Accurate-Mass TOF mass spectrometer (Agilent Technologies, Wilmington, DE, USA) with a dual electrospray ionization source from a methanol solution. The following parameters were used: nitrogen flow 5 L min^–1^, gas temperature 325 °C, nebulizer 45 psi, skimmer 65 V, and fragmentor 50 and 100 V. IR spectra were recorded on a Bruker Tensor 27 FTIR spectrometer with a Bruker Platinum ATR system. The solution NMR spectra were recorded on a Bruker Avance III 300 NMR spectrometer at frequencies of 300.1 MHz for ^1^H and 121.5 MHz for ^31^P in 5 mm NMR tubes. CD_3_OD and C_6_D_6_ were used as an internal lock. The spectra were referenced to the residual proton signal of CHD_2_OD (3.33 ppm) and C_6_HD_5_ (7.16 ppm), while the ^31^P spectra were referenced to 85% H_3_PO_4_ (0 ppm). Hydrogen temperature-programmed reduction (H_2_-TPR) was carried out on AutoChem II-2920 equipment (Micromeritics, Atlanta, GA, USA). Before each H_2_-TPR experiment, the sample (0.1 g) was pretreated in Ar (50 mL min^–1^) at 300 °C for 30 min. The sample was cooled to 50 °C in the same atmosphere and then reduced in a hydrogen–argon mixture (10 mol % H_2_/Ar) at a flow of 50 mL min^–1^ and constant heating rate of 10 °C min^–1^ up to 500 or 700 °C and held at this temperature for 30 min. The water vapor formed during the TPR measurements was captured in a cold trap. Correction based on the signal of the neat silica support (Aerosil 300) was used for the evaluation of the number of reducible species.

### Ligand and Complex Synthesis

**Figure 1:**
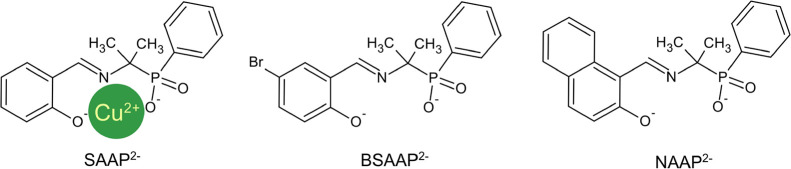
Schematic representation of the L^2–^ ligands used in this study. The green circle schematically depicts the copper(II) atom situated in the coordination pocket of the SAAP^2–^ ligand.

Detailed synthetic procedures of (2-{[(E)-(2-hydroxyphenyl)methylidene]amino}propan-2-yl)phenylphosphinate (HSAAP^–^), (2-{[(E)-(5-bromo-2-hydroxyphenyl)methylidene]amino}propan-2-yl)phenylphosphinate (HBSAAP^–^), and (2-{[(E)-(2-hydroxynaphthalen-1-yl)methylidene]amino}propan-2-yl)phenylphosphinate (HNAAP^–^) sodium salts (Figure 1) are described in Supporting Information. Ligands HSAAP^–^, HBSAAP^–^, and HNAAP^–^ were deprotonated and used to prepare copper phosphinate complexes {Cu(SAAP)}_*n*_, (**1**), [Cu_6_(BSAAP)_6_] (**2**), and [Cu_3_(NAAP)_3_] (**3**). Synthesis details and characterization are described in Tables S1–S3. Copper phosphinate complexes **1**–**3** were used as precursors for catalyst preparation (see below).

### Catalyst Preparation

**CuP-1**–**3** and **CuP-3-TEP** (10-fold loading of Cu) catalysts were prepared by the wet impregnation of the corresponding complex **1**–**3** on the commercial SiO_2_ support (Aerosil 300) from MeOH solution (50 cm^3^). After Aerosil was added to a clear solution of a complex, the suspension was sonicated for 5 min for homogenization and then evaporated on a rotary evaporator. The well-dried, homogeneously green sample was calcined in air for 10 h at 500 °C, resulting in a light-blue product. The weight of the complex and support was calculated to obtain 2.5 wt % Cu loading for **CuP-1**–**3** and 25 wt % Cu loading for **CuP-3-TEP** (masses used in the preparation are summarized in Table S4). The **Cu-DI** benchmark catalyst was prepared by the previously reported procedure.^20^ Cu(NO_3_)_2_·2.5H_2_O (91.5 mg, 0.393 mmol) was dissolved in water (10 cm^3^) and mixed with silica (Aerosil 300, 1.0 g) to form a paste. The sample was dried in an oven at 70 °C with occasional mixing. The dried catalyst was ground and calcined at 500 °C for 5 h. The **CuP-Y** benchmark catalyst was prepared according to the previously reported procedure^102^ by the dry impregnation method. Cu(NO_3_)_2_·2.5H_2_O (188 mg, 0.808 mmol) was dissolved in water (20 cm^3^) and mixed with silica (Aerosil 300, 2.0 g) to form a paste. The sample was dried in an oven at 100 °C with occasional mixing. Then a solution of 85% H_3_PO_4_ (0.093 g, 0.807 mmol) in 10 cm^3^ of water was added, and the sample was mixed well and dried at 100 °C with occasional mixing. The dried catalyst was ground and calcined at 500 °C for 10 h. The **CuP-P** benchmark catalyst was prepared by the addition of a solution of (NH_4_)_2_HPO_4_ (0.053 g, 0.401 mmol) in 25 cm^3^ of water to a suspension of silica (Aerosil 300, 1.0 g) in a solution of Cu(NO_3_)_2_·2.5H_2_O (0.094 g, 0.404 mmol) in methanol (25 cm^3^)_._ The mixture was then sonicated for a few minutes to ensure proper homogenization. After sonication, the suspension was dried using a rotary evaporator and then calcined at 500 °C for 10 h.

### Catalytic Reactor Details

For catalysis, a fixed-bed catalytic reactor was used. Gas chromatography with a flame ionization detector was used to determine the catalytic activity. Catalytic tests were performed at 325 °C for up to 50 h. The effluent gas analysis was carried out by an HP 6890 Gas Chromatograph equipped with a flame ionization detector and a Thermo scientific TG-BOND U column (length of 30 m, internal diameter of 0.32 mm, and film thickness of 10 μm). Calcined catalysts (200 mg) with selected grain size (0.2–0.4 mm) were diluted with glass beads (0.5–1 mm) to a constant volume. The void space in the reactor tube was filled with glass beads. Before the reaction, the catalysts were pretreated in situ by feeding hydrogen (10 vol % H_2_ in N_2_) for 1 h at 400 °C (Cu reduction). During all processes, nitrogen was used as carrier gas (50 cm^3^ min^–1^); ethanol was fed by a NE-300 syringe pump with WHSV 2.37 h^–1^ (7.7 mol % ethanol in N_2_). Pentane was added as the internal standard (5% molar concentration in ethanol feed). The tests were carried out at atmospheric pressure.

## Results and Discussion

### Cu(II) Phosphinate Complex Synthesis and Structure

The reactions between the ligands SAAP^2–^, BSAAP^2–^, and NAAP^2–^ (Figure 1) in the methanolic solution with the equimolar amount of the Cu(NO_3_)_2_·2.5H_2_O and sodium hydroxide lead to three new Cu(II) phosphinate complexes. The obtained compounds were isolated from the byproducts by the dissolution of the dried reaction mixture in THF, filtration, drying, and crystallization of the pure products from acetonitrile solutions. Detailed synthetic procedures and the characterization of the ligands and complexes are described in the Supporting Information.

The single crystals were obtained for all three Cu(II) phosphinate complexes, and molecular structure models were obtained by single-crystal X-ray diffraction. Complex **1** crystallizes as a 1D polymer with the repeating formula unit {Cu(SAAP)}_*n*_ in the monoclinic *P*2/*c* space group, while compounds **2** and **3** crystallize in the triclinic *P*1̅ space group and remain in the molecular form with the formulas [Cu_6_(BSAAP)_6_] and [Cu_3_(NAAP)_3_], respectively. The main crystallographic and refinement parameters are summarized in Table S1. For all three structures, the primary trend is that Cu^2+^ cations are coordinated in the ONO coordination pocket of the ligands (Figure 1).

**Figure 2:**
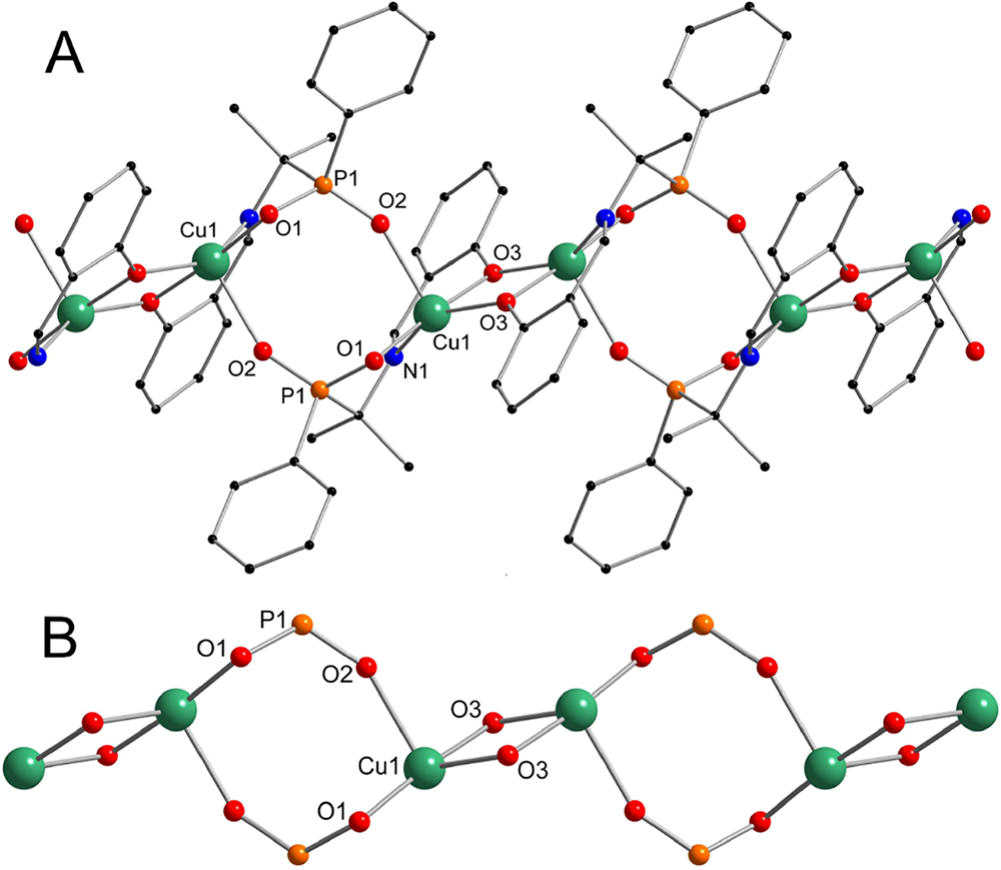
Ball and stick representation of the structure of **1**. The complete chain structure (A) and phosphinate cores connected by the phenolic oxygen bridges (B). Color code: Cu green, P orange, O red, N blue, and C black. Hydrogen atoms have been omitted for the sake of clarity.

Further coordination and final arrangement of the Cu(II) phosphinate complexes are driven by steric differences in the ligand molecules. The polymeric structure of **1** could be described as binuclear units Cu_2_(SAAP)_2_, consisting of two Cu(SAAP) moieties interconnected to the cycle by the second phosphinic oxygen (not involved in the ONO coordination pocket) with a formation of two Cu–OPO–Cu bridges. The units are connected to the polymer chain by mutual coordination of copper(II) atoms by phenolic oxygen atoms. All copper(II) atoms in the structure of **1** are crystallographically equivalent and five-coordinated by the ONO pocket atoms, one phosphinic, and one phenolic oxygen atom from two other ligands, as depicted in Figure 2 (coordination polyhedra will be discussed below). The closest distance between the copper(II) atoms is equal to 3.059 Å in the phenoxy-bridged Cu_2_O_2_ moiety.

**Figure 3:**
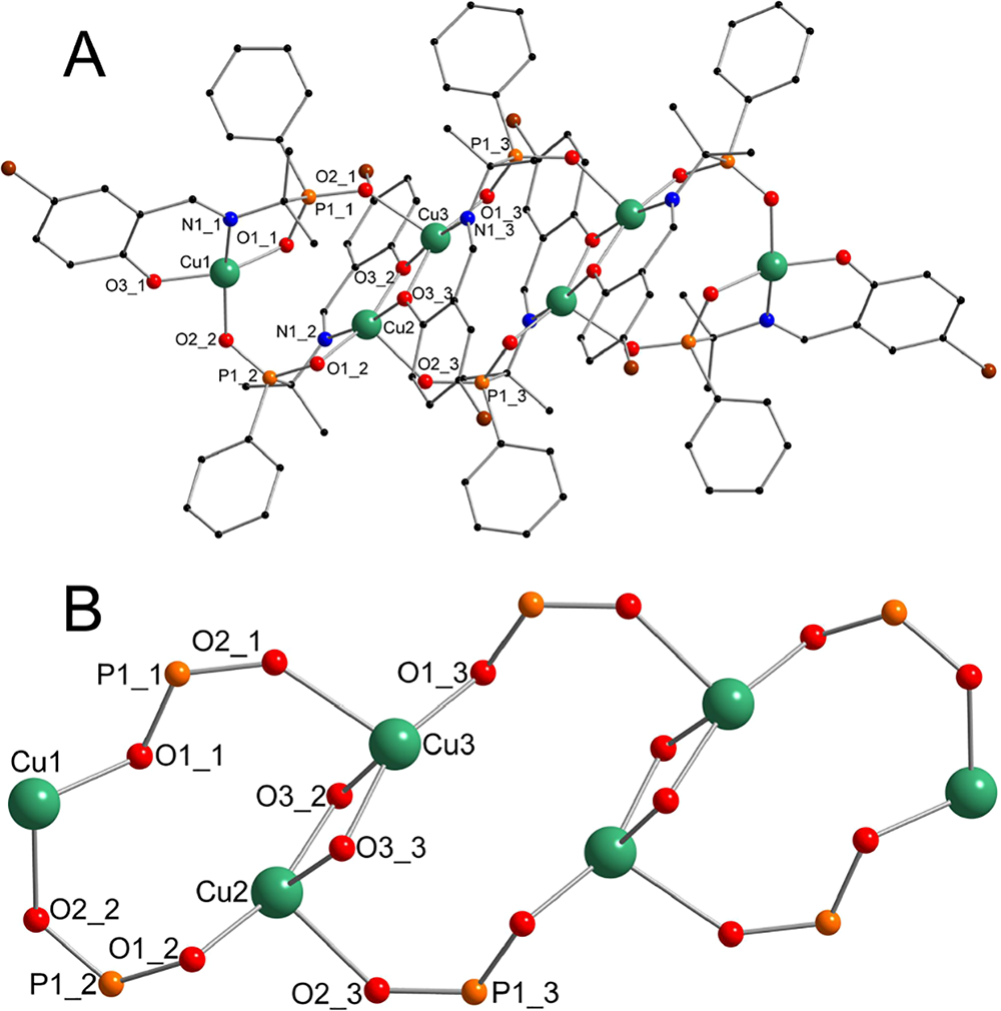
Ball and stick representation of the structure of **2**. The models of the molecule (A) and the core only (B). Color code: Cu green, P orange, O red, N blue, C black, and Br brown. Hydrogen atoms were omitted for the sake of clarity.

In the case of **2**, the formation of the centrosymmetric hexanuclear complex was observed. The whole molecular motive is more complicated than that of other complexes (Figure 3). Three independent copper(II) atom centers of the molecule are four- (Cu1) and five-coordinated (Cu2 and Cu3). The coordination environments of copper(II) atoms are based on an ONO coordination pocket and are completed by a phosphinic oxygen atom of another ligand (Cu1) and one phosphinic and one phenolic oxygen atom from two other ligands (Cu2 and Cu3), as depicted in Figure 3. All copper(II) atoms are situated on the same plane. The closest distance of 3.019 Å was observed between Cu2 and Cu3 atoms in phenoxy-bridged Cu_2_O_2_ moieties similar to that of copper phosphinate **1**.

**Figure 4:**
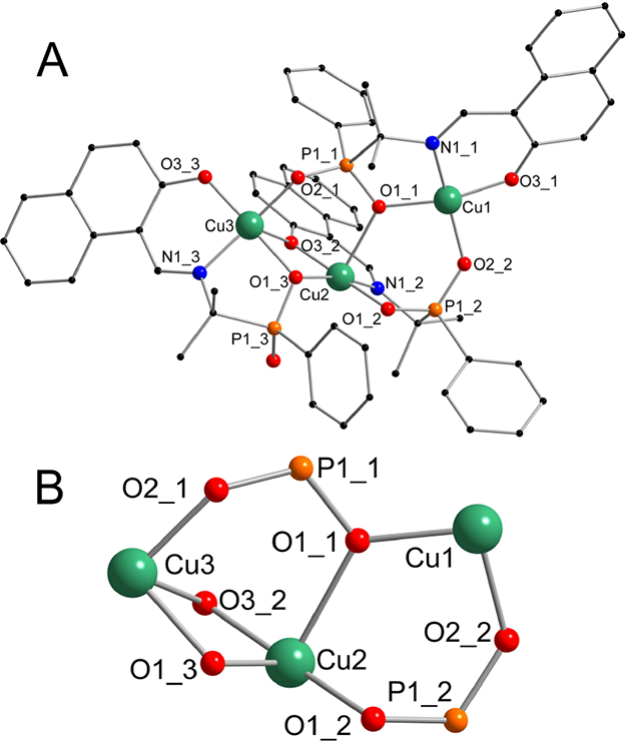
Ball and stick representation of the structure of **3**. The model of the molecule (A) and the core only (B). Color code: Cu green, P orange, O red, N blue, and C black. Hydrogen atoms were omitted for the sake of clarity.

The structure of trinuclear molecular complex **3** is similar to the structure of **2** if it is cut in half. In correspondence to **2,** Cu1 is four-coordinated, while Cu2 and Cu3 are five-coordinated. The main difference is that in **2,** the Cu2 and Cu3 atoms are connected by two phenolic oxygen bridges, while in **3**, they are connected by one phenolic and one phosphinic oxygen bridge. This, combined with the steric effect of the ligand, results in a larger distance between Cu2 and Cu3 atoms, reaching 3.115 Å (Figure 4).

Selected bond lengths in the Cu polyhedra are summarized in Table S2. Continuous shape measures (CShM)^113−116^ of the copper(II) atoms polyhedra for the formed complexes **1**–**3** showed that the geometry of five-coordinated polyhedra is closer to the square pyramidal in all cases than to trigonal bipyramidal with the distortion values in the range of 0.726–2.550 (see Table S3). The polyhedra of the four-coordinated Cu^2+^ cations in complexes **2** and **3** are closer to square than tetrahedral geometry (see Table S3).

The molecular nature of the dissolved complexes **1**–**3** used in the catalyst preparation was confirmed through the study of their methanolic solutions by ESI-MS spectroscopy. The spectra were recorded in positive and negative modes under different conditions. The coordination polymer **1** exhibited the presence of small molecular clusters with nuclearity ranging from 2 to 5 when the fragmentor voltage was set to 100 V. When 50 V was applied, the most intense peaks corresponded to four and five nuclear fragments (Figures S1 and S2). The spectra of the hexanuclear complex **2**, recorded with fragmentor voltages of 50 and 100 V, displayed similar patterns, showing the parent peak [Cu_6_(BSAAP)_6_ + H]^+^ with a relative intensity of ∼4%. The presence of smaller fragments containing 5 to 2 Cu atoms with higher intensities confirms the fragmentation of the molecule during the ionization process (Figures S3 and S4). The spectra of trinuclear complex **3**, recorded with a fragmentor voltage of 100 V, predominantly exhibited the molecular parent peaks ([Cu_3_(NAAP)_3_ + H]^+^ and [Cu_3_(NAAP)_3_ + Na]^+^) when a lower concentration of the sample was used (Figure S5). Furthermore, alongside the parent peaks, the spectra displayed aggregation into larger species containing 4 and 5 Cu atoms when a higher concentration of the sample was employed (Figure S6). The spectra recorded in negative ion mode do not provide significant information, as larger fragmentation occurs for all complexes and the peaks of lower nuclearity are predominantly observed (Figures S2, S4, and S6).

The behavior of Cu(II) phosphinate complexes **1**–**3** upon heating was analyzed by the TG/DSC method (Figures S7–S9). Complexes **1** and **3** showed thermal stability up to 250 °C, while complex **2** was stable practically to 300 °C. Above these temperatures, continuous weight losses were observed until 800 °C. The residual masses at 1000 °C were 40.21, 28.90, and 33.91 wt % for **1**–**3**, respectively.

Complexes **1**–**3** calcined at 500 °C (5 °C min^–1^, air, 10 h) and 1000 °C (5 °C min^–1^, air, no dwell) were studied by powder X-ray diffraction analysis (PXRD). The predominant phase observed in all samples calcined at 500 °C is copper diphosphate Cu_2_P_2_O_7_ (PDF: 00–044–0182)^117,118^ (Figure S10). Additionally, the presence of an intense diffraction around 10° 2Θ points to the presence of an additional phase of hydrated copper diphosphate Cu_2_P_2_O_7_·3H_2_O (PDF: 00–051–0202)^119^ (Figure S10). Partial hydration could occur due to the samples being exposed to atmospheric moisture during the time between calcination and PXRD analysis. The primary phase observed in samples calcined at 1000 °C remains Cu_2_P_2_O_7_ (PDF: 00–044–0182)^117,118^ (Figure S11). However, there is an increasing presence of the Cu_3_(PO_4_)_2_ phase (PDF: 00–080–0992)^120^ content in the succession of calcined complexes from **1** to **3** (Figure S11).

The absorption bands typical for phosphate group vibrations were observed in the IR spectra of Cu(II) phosphinate complexes after calcination at 500 °C for 10 h. Also, no absorption bands of the C–H stretches were observed in the 2800–3000 cm^–1^ region (Figure S12). Therefore, the temperature of 500 °C and 10 h calcination time were applied in the catalyst preparation (see the Synthesis and Characterization of Cu-Phosphate/SiO2 Catalysts section).

### Synthesis and Characterization of Cu-Phosphate/SiO_2_ Catalysts

Three Cu(II) phosphinate complexes **1**–**3** were used as precursors for copper and phosphorus deposition on porous commercial SiO_2_ by wet impregnation to prepare the **Cu-phosphate/SiO**_**2**_ catalysts (**CuP-1**–**3**). A benchmark sample without phosphorus was prepared by the dry impregnation method similar to a promising Cu/SiO_2_ ethanol dehydrogenation catalyst working at 325 °C (**Cu-DI**).^20^ A benchmark catalyst containing phosphorus was prepared using two methods. The **CuP-Y** catalyst was prepared using the method described by Yamamoto et al.,^102^ employing the dry impregnation technique. On the other hand, the **CuP-P** catalyst was prepared through precipitation of CuHPO_4_ in the silica suspension. Experimental loadings of Cu and P in the catalysts after calcination are summarized in Table 1. Cu contents in the **Cu-phosphate/SiO**_**2**_ catalysts were in the range of 1.76 to 2.33 wt %. The atomic Cu:P ratios were close to 1:1 in all three catalysts and thus followed the atomic Cu:P ratios in the starting Cu(II) phosphinate precursors. The benchmark catalysts containing phosphorus exhibited the same Cu:P ratio (close to 1:1).

**Table 1:** Experimental Cu and P Loadings in Catalysts (ICP-OES)

**sample**	**precursor**	**Cu** loading [wt %]	**P** loading [wt %]	**Cu:P mol ratio**
**CuP-1**	{Cu(SAAP)}_*n*_ (**1**)	1.79	0.93	0.93
**CuP-2**	[Cu_6_(BSAAP)_6_] (**2**)	2.33	1.13	1.0
**CuP-3**	[Cu_3_(NAAP)_3_] (**3**)	1.76	0.85	1.0
**Cu-DI**	Cu(NO_3_)_2_·2.5H_2_O	2.42		
**CuP-Y**	Cu(NO_3_)_2_·2.5H_2_O + H_3_PO_4_	2.19	1.11	0.96
**CuP-P**	Cu(NO_3_)_2_·2.5H_2_O + (NH_4_)_2_HPO_4_	2.25	1.12	0.97

All catalysts were prepared using the same silica support Aerosil 300 (284 m^2^ g^–1^, 1.55 cm^3^ g^–1^, isotherm shown in Figure S13). The porosity of **Cu-phosphate/SiO**_**2**_ samples was very similar to that of the catalyst support and the **Cu-DI**, **CuP-Y**, and **CuP-P** benchmark catalyst (Table 2). Surface areas (SA) ranged from 250 to 282 m^2^ g^–1^, pore volumes (*V*_total_) from 1.04 to 1.14 cm^3^ g^–1^_,_, and average pore diameters (*d*_pore_) from 15 to 18 nm. The N_2_ adsorption and desorption isotherms are shown in Figure S14.

**Table 2:** Comparison of the Prepared Catalysts by N_2_ Porosimetry

**sample**	**SA** [m^2^ g^–1^]	*V*_**total**_ [cm^3^ g^–1^]	***d***_**pore**_**[nm]**^a^
**Aerosil 300**	284	1.55	22
**CuP-1**	258	1.14	18
**CuP-2**	282	1.04	15
**CuP- 3**	250	1.10	18
**Cu-DI**	245	1.45	24
**CuP-Y**	252	1.40	22
**CuP-P**	240	1.57	26

a Estimated by .

**Figure 5:**
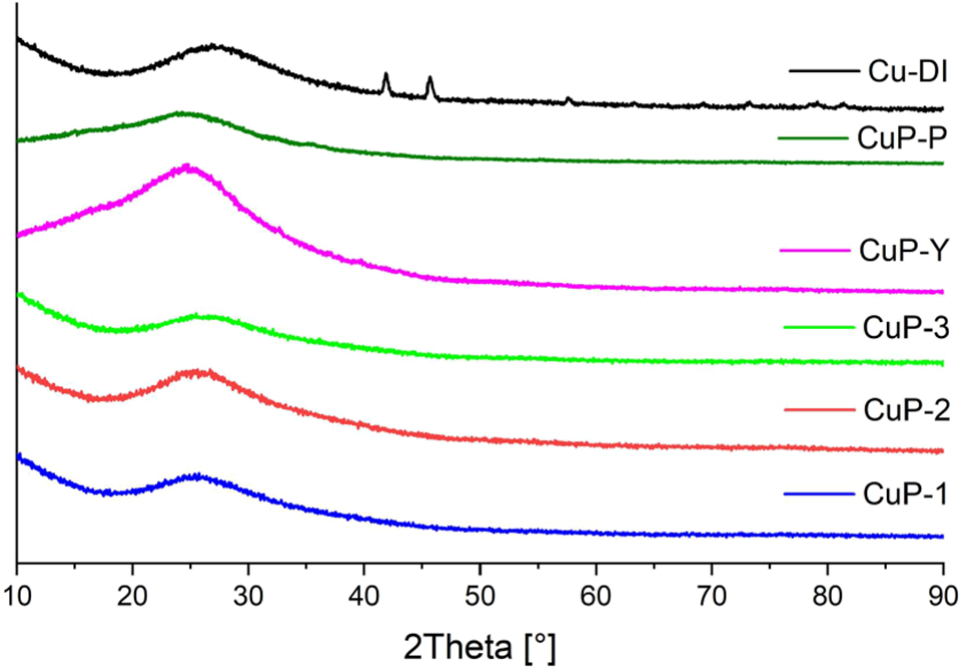
Comparison of the X-ray diffractograms of the fresh catalysts (calcined under an ambient atmosphere). **Cu-phosphate/SiO**_**2**_, **CuP-Y**, and **CuP-P** samples are X-ray amorphous, and diffractions of CuO were observed (PDF: 00–048–1548)^121^ for **Cu-DI**.

Powder X-ray diffraction analysis showed that all **Cu-phosphate/SiO**_**2**_ catalysts prepared from the Cu(II) phosphinate complexes and benchmark catalysts containing phosphorus (**CuP-Y**, **CuP-P**) were X-ray amorphous after calcination in the ambient atmosphere (Figure 5). On the contrary, the sample prepared by dry impregnation of copper nitrate (**Cu-DI**) exhibited diffractions corresponding to copper(II) oxide (PDF: 00–048–1548).^121^ According to the Debye–Scherrer equation, the crystallite size was estimated to be 22 nm.^20^

**Figure 6:**
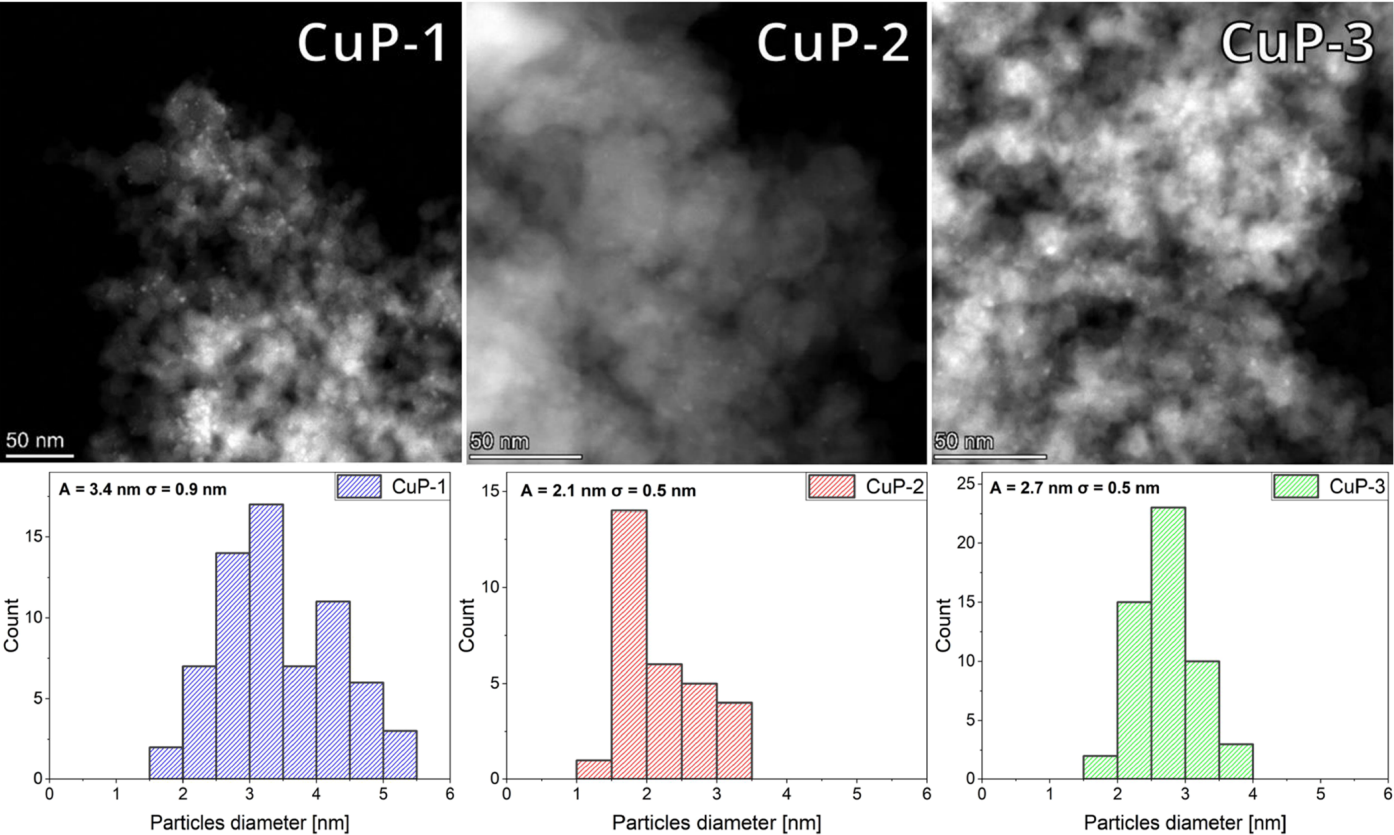
STEM micrographs of samples **CuP-1**–**3** after calcination (top) and comparison of their particle size distribution histograms (below).

**Figure 7:**
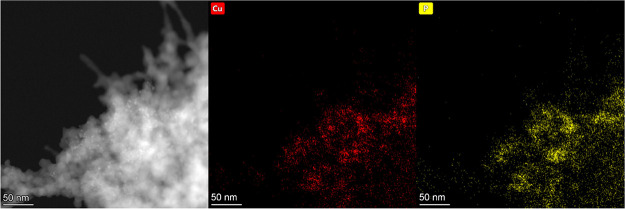
STEM micrograph of **CuP-1** after calcination and STEM-EDS elemental micrograph survey of copper (red) and phosphorus (yellow).

STEM-EDS analysis of the **Cu-phosphate/SiO**_**2**_ samples in all cases (**CuP-1**–**3**) displayed small and uniform particles (Figure 6). The sample **CuP-1** prepared from the polymer precursor {Cu(SAAP)}_*n*_ showed the largest nanoparticles (A̅ = 3.4 nm; σ = 0.9 nm) of the copper phosphate phase with a broader size distribution. Thus, the polymeric nature of precursor **1** possibly leads to a slight increase in particle size in comparison with molecular ones ([Cu_6_(BSAAP)_6_] and [Cu_3_(NAAP)_3_]). It should be noted that polymeric complex **1** can dissociate in solution into fragments with varying nuclearity (ESI-MS, see discussion above), potentially resulting in a broader size distribution of the resulting nanoparticles. Based on the graphic analysis of the STEM micrograph survey, particles in **CuP-2** prepared from [Cu_6_(BSAAP)_6_] exhibited the NP size of A̅ = 2.1 nm and σ = 0.5 nm. On the other hand, the **CuP-3** catalyst prepared from [Cu_3_(NAAP)_3_] showed slightly larger particles (A̅ = 2.7 nm; σ = 0.5 nm), despite the molecules of **3** (precursor for **CuP-3**) being smaller than **2**. According to STEM-EDS elemental mapping, these particles consist of Cu and P (Figure 7). Thus, it can be inferred that the particles observed in STEM-EDS micrographs present an X-ray amorphous copper phosphate phase (see below for the PXRD, TG/DSC, IR, and XPS spectroscopy study). The formation of the copper phosphate phase comes from the original application of copper phosphinates as molecular precursors.

### Catalysis

**Figure 8:**
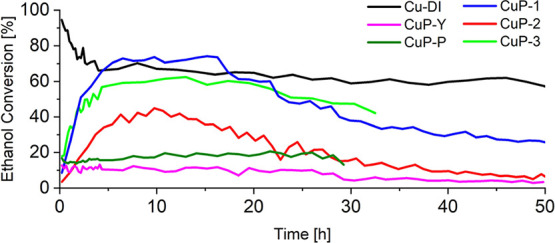
Evolution of ethanol conversion over time: Comparison of the benchmark catalysts (**Cu-DI**, **CuP-Y**, and **CuP-P**) with catalysts prepared from Cu(II) phosphinate complexes.

**Figure 9:**
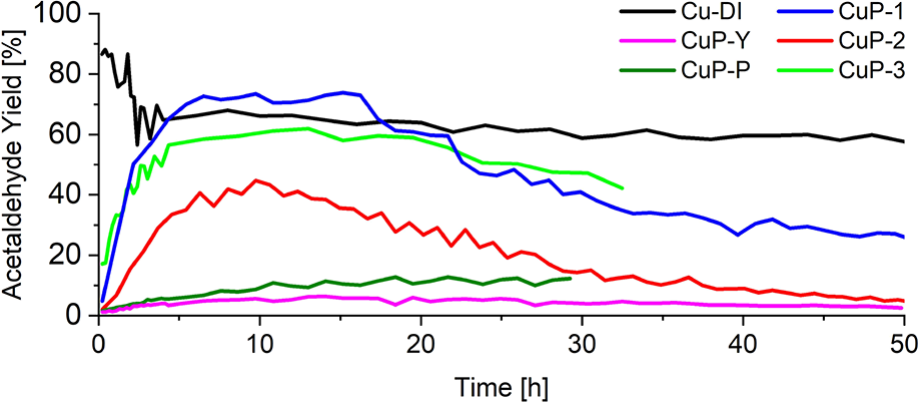
Acetaldehyde yield over the **Cu-phosphate/SiO**_**2**_ catalysts and comparison with the **Cu-DI**, **CuP-Y**, and **CuP-P** benchmark samples.

Ethanol conversion over **CuP-1**–**3**, **CuP-Y**, **CuP-P**, and **Cu-DI** catalysts at 325 °C is shown in Figure 8. A remarkable difference in catalyst performance with time was observed between the **Cu-phosphate/SiO**_**2**_ catalysts and phosphorus-free **Cu-DI** (Figures 8 and 9). The sample prepared by the dry impregnation method (**Cu-DI**) achieved ethanol conversion up to 95% at the beginning of the catalytic process. The sample showed rapid deactivation during the first 5 h of the measurement (ethanol conversion dropped to ca. 70%). Afterward, the deactivation was much slower until the end of the catalytic experiment, with ethanol conversion being ca. 60% after 50 h. This behavior is in good agreement with numerous studies that have pointed out that the copper-based catalysts supported on silica often suffer from deactivation.^7,16−18,122,123^

In contrast, the samples prepared from Cu(II) phosphinate complexes showed an initial increase in catalytic activity during the first 1.6–8 h before reaching the maximum ethanol conversion (3.3–10 h). The maximum ethanol conversion reached ca. 73% for **CuP-1**, ca. 40% for **CuP-2**, and ca. 60% for **CuP-3**, as shown in Figure 8. The ethanol conversion of **CuP-1** and **CuP-3** at the top of catalytic activity was comparable to **Cu-DI** at similar TOS (∼70%). After 6–13 h of the catalytic experiment, the course of this reaction changed, and the catalysts gradually began to deactivate, faster than **Cu-DI**. Additional catalytic experiments and analyses (ICP-OES, XPS, and STEM-EDS) were performed to better understand the catalytic behavior of the **Cu-phosphate/SiO**_**2**_ catalysts. These results are thoroughly discussed in the Changes to the Cu-Phosphate/SiO_2_Catalysts during Time-on-Stream section.

A similar behavior (i.e., an increase of the catalytic activity at the beginning and then decline) was observed in phosphorus-containing **CuP-Y** and **CuP-P** benchmark catalysts. However, the activation rate was much slower than in **Cu-phosphate/SiO**_**2**_ samples and the maximum ethanol conversion was achieved after 20–30 h. Also, the catalytic activity of **CuP-Y** and **CuP-P** catalysts was significantly lower compared to **Cu-phosphate/SiO**_**2**_. The highest ethanol conversion was 13% for **CuP-Y** and 20% for **CuP-P**.

The acetaldehyde selectivity significantly varied among the samples. Both **Cu-phosphate/SiO**_**2**_ and phosphate-free **Cu-DI** catalysts exhibited very high selectivity to acetaldehyde (≥95% for **Cu-DI** and ≥98% for **Cu-phosphate/SiO**_**2**_) with a carbon balance fluctuating around 95%. Therefore, the acetaldehyde yields closely followed ethanol conversion for **CuP-1–3** and **Cu-DI**. (Figures 8 and 9). On the contrary, a low selectivity to acetaldehyde was observed for phosphorus-containing benchmark catalysts (average selectivity 53% for **CuP-Y** and 43% for **CuP-P**). The ethanol dehydration to ethylene and diethyl ether was the reason for the low selectivity to acetaldehyde.

The surface Cu concentration was evaluated by XPS in all samples before and after catalytic experiments to check a possible correlation between the catalytic activity and the number of surface Cu species (Table S5). The surface Cu concentration in **CuP-1–3** is low (0.17–0.37 wt %), slightly changes with TOS, and does not correlate with the observed catalytic activity. The variations in surface Cu content are caused by the limits of XPS analysis. Cu surface content (∼0.1 at. %) is close to the detection limit,^124^ and therefore quantification can be problematic. It is also possible that copper in interparticle voids is active in catalysis, but it is not “visible” via the XPS method.

Importantly, the low active phosphorus-containing benchmarks **CuP-P** and **CuP-Y** were not fully reduced to Cu^0^/Cu^+^ species after catalytic experiments in contrary to **CuP-1–3**: the typical peak at ∼935 eV and satellite peak at ∼944 eV for Cu^2+^ was still observed in spent samples (see Changes to the Cu-Phosphate/SiO_2_Catalysts during Time-on-Stream section). The impossibility of fully reducing copper in **CuP-P** and **CuP-Y** (and thus converting it to catalytically active Cu^0^ and Cu^+^ species according to literature)^125^ might be connected with the phosphorus presence and will be discussed in the Changes to the Cu-Phosphate/SiO_2_Catalysts during Time-on-Stream section. Importantly, it might be one of the reasons implying a low activity of phosphorus-containing benchmark catalysts. Another reason for the low catalytic activity of **CuP-Y** and **CuP-P** might be a significant sintering of Cu particles. XRD showed intense diffractions of metallic Cu after catalytic experiments (while samples were fully XRD amorphous before catalysis; see Changes to the Cu-Phosphate/SiO_2_Catalysts during Time-on-Stream section). In agreement, STEM-EDS analysis displayed the presence of sintered particles after catalytic tests for both **CuP-Y** and **CuP-P** (∼100–500 nm; Figures S15 and S16).

The catalytic activity of the **Cu-phosphate/SiO**_**2**_ samples seemingly displayed a size dependence. Surprisingly, the most active catalyst was **CuP-1** possessing the largest particles (A̅ = 3.4 nm; σ = 0.9 nm), while the least active was **CuP-2** with the smallest particles (A̅ = 2.1 nm; σ = 0.5 nm). The particle size distribution was narrow in all three **Cu-phosphate/SiO**_**2**_ catalysts and probably did not play a crucial role in catalysts performance. Also, the surface areas of the **Cu-phosphate/SiO**_**2**_ samples were similar, in the range of 250–282 m^2^ g^–1^, and probably did not significantly affect catalytic activity. Finally, sample **CuP-2** exhibited the highest copper loading (2.33 wt %), but it displayed the lowest catalytic activity compared to **CuP-1** and **CuP-3,** with copper loading of 1.79 and 1.75 wt %, respectively.

Notably, the particle size was controlled by the Cu phosphinate complex used in the catalyst preparation, and thus, the precursor choice influenced the catalytic activity of the final material. The trend in catalytic activity seems to disagree with the reports describing ethanol dehydrogenation over Cu NPs, where the smaller particles usually provide higher catalytic performance.^126^ However, a thorough characterization is needed to understand better the catalytic properties of **Cu-phosphate/SiO**_**2**_ materials (see the Changes to the Cu-Phosphate/SiO_2_Catalysts during Time-on-Stream section).

Table 3 shows a comparison of previously reported catalysts and the most active newly developed **Cu-phosphate/SiO**_**2**_ catalyst **CuP-1** from this study presented at its maximum catalytic activity. **CuP-1** shows promising activity compared to other Cu-based catalysts. Despite the relatively low Cu loading of 1.79 wt %, **CuP-1** can achieve high acetaldehyde productivity with high selectivity. However, its stability needs further improvement.

**Table 3:** Comparison of Acetaldehyde Productivity at 325 °C of **Cu-phosphate/SiO**_**2**_ Catalyst **CuP-1** (at the Maximum Activity) with the Data Presented in the Literature (no Phosphorus in the Referenced Materials)

**sample**	**Cu [wt %]**	**WHSV [h**^**–1**^**]**	***T* [°C]**	**conversion [%]**	**acetaldehyde selectivity [%]**	**acetaldehydeproductivity** [g g^–1^ h^–1^]
**CuP-1**^This work^	1.79	2.37	325	73	≥98	1.70
**Cu/SiO**_**2**_(123)	25	2.37	300	75	94	1.67
**Cu/SiO**_**2**_**-AE**(16)	2.7	3.16	300	98	≥99	3.07
**Cu/SiO**_**2**_(20)	2.42	4.73	325	50	95	2.24
**Cu/β Zeolite**(17)	5	1	325	91	79	0.72

### Changes to the Cu-Phosphate/SiO_2_ Catalysts during Time-on-Stream

Additional analyses were performed to understand the peculiar catalytic performance of the **Cu-phosphate/SiO**_**2**_ samples. The Cu:P ratio was ex-situ analyzed by ICP-OES analysis for **CuP-1** at different stages of the catalyst life: (i) calcined in ambient atmosphere, (ii) after H_2_ treatment, (iii) at the maximum of ethanol conversion (i.e., after 7 h of TOS), and (iv) at the end of the catalytic experiment (after more than 50 h of TOS; Table 4). It can be seen that the Cu:P ratio did not change after H_2_ treatment in comparison to the fresh calcined sample; in both cases, the ratio stayed close to 1 according to ICP-OES. During the ethanol dehydrogenation process, the amount of phosphorus steadily declined; the Cu:P ratio increased to 1.3 for the sample at the top of catalytic activity and to 1.9 at the end of the catalytic test (Table 4). Interestingly, the decrease in total phosphorus content by ICP-OES was confirmed also for phosphorus-containing benchmark catalysts **CuP-Y** and **CuP-P**. However, the phosphorus decline in sample **CuP-Y** took place to a lesser extent in comparison to **CuP-1**.

**Table 4:** ICP-OES Analysis of **CuP-1** at the Different Stages of the Catalyst Life

**catalyst**	**sample**	**Culoading** [wt %]	**Ploading** [wt %]	**Cu:P mol ratio**
**CuP-1**	Calcined	1.79	0.93	0.93
	H_2_ treated	1.75	0.92	0.93
	At the top of catalytic activity	1.77	0.66	1.3
	Spent catalyst	1.83	0.47	1.9
**CuP-Y**	Spent catalyst	2.83	0.97	1.4
**CuP-P**	Spent catalyst	1.68	0.65	1.3

To investigate the observed phosphorus leaching over time-on-stream (TOS) in more detail, a new catalyst was prepared using complex **3** with approximately 10-fold Cu content (**CuP-3-TEP**). Subsequently, an additional catalytic experiment was conducted, and the products were collected in an ice-cooled trap (for details, see Supporting Information). The volatile compounds were then evaporated using a rotary evaporator, and the residue was analyzed by applying ^1^H and ^31^P {^1^H} NMR in C_6_D_6_ (Figure S17). The NMR spectra revealed the presence of triethyl phosphate (TEP) as the only phosphorus-containing compound. Thus, the ethanol reacts with the phosphates and converts them to TEP. The molecules of TEP are then eluted in the gas stream, and the phosphorus content in the **Cu-phosphate/SiO**_**2**_ catalysts steadily declines with TOS.

**Figure 10:**
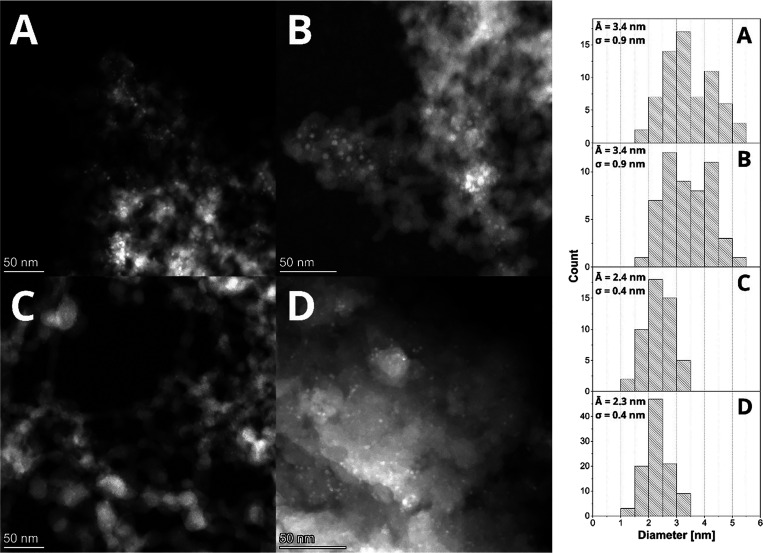
STEM micrographs of catalyst **CuP-1** after calcination (A), after H_2_ treatment (B), at the point of the highest catalytic activity (C), and after the whole catalytic cycle (D).

Nanoparticle sizes at the different stages of the **CuP-1** catalyst lifetime were monitored by ex-situ STEM analyses (Figure 10). Particles in the sample after H_2_ treatment (A̅ = 3.4 nm; σ = 0.9) were similar compared to the fresh catalyst, agreeing with no dramatic changes (Figure 10A,B). The increase in the catalyst activity was followed by a significant nanoparticle size decrease (A̅ = 2.4 nm; σ = 0.5 nm) (**CuP-1** at the maximum catalytic activity; Figure 10C). STEM characterization of the spent catalyst (Figure 10D) revealed a continuous decrease in the nanoparticle size and particle size distribution.

**Figure 11:**
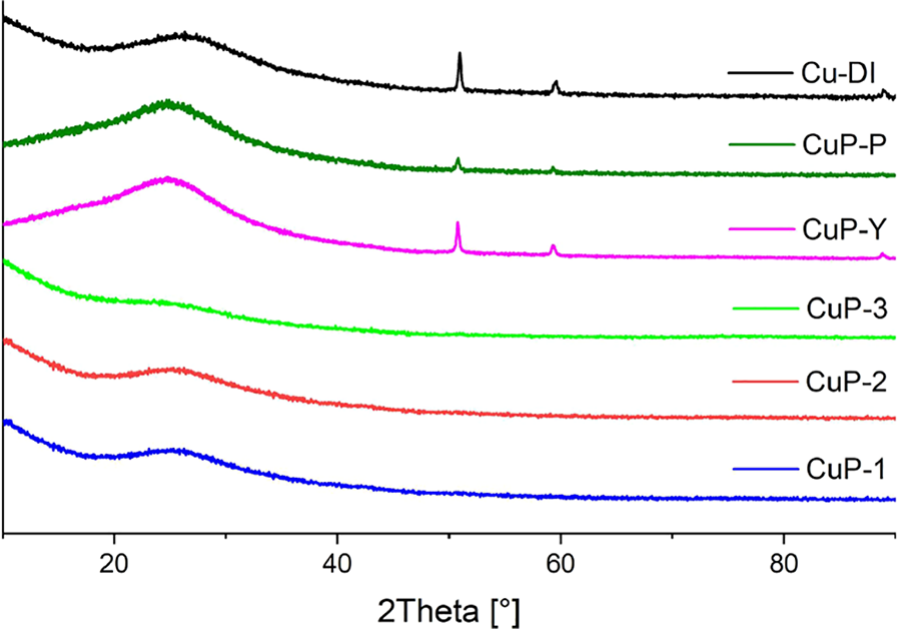
XRD diffraction patterns of spent catalysts; **Cu-phosphate/SiO**_**2**_ samples remained amorphous, and diffractions of metallic copper were observed (PDF: 00–004–0836)^127^ for **CuP-Y, CuP-P,** and **Cu-DI**.

The **Cu-phosphate/SiO**_**2**_ catalysts remained X-ray amorphous after the whole catalytic cycle (Figure 11). In contrast, the **Cu-DI**, **CuP-Y**, and **CuP-P** were reduced to metallic copper (PDF: 00–004–0836)^127^ after the catalytic reaction. Moreover, as we reported recently, **Cu-DI** was reduced to metallic copper after H_2_ treatment. Crystallite sizes estimated by the Debye–Scherrer equation for **Cu-DI** after both H_2_ treatment and the whole catalytic cycle remained similar (ca. 22 nm).^20^

**Figure 12:**
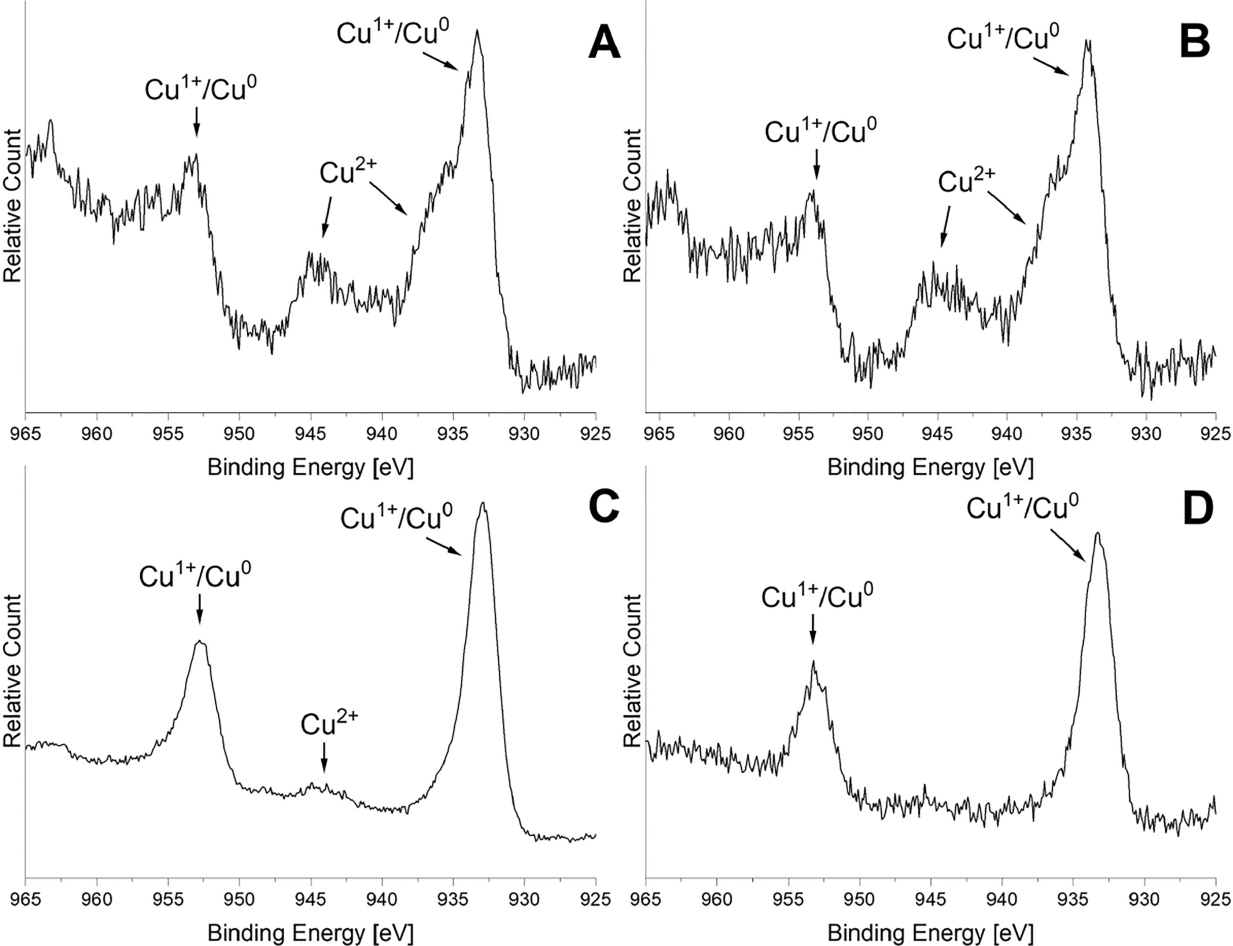
High-resolution XPS spectra (Cu 2p) recorded for catalyst **CuP-1** after calcination (A), after H_2_ treatment (B), at the point of the highest catalytic activity (C), and after the entire catalytic cycle (D).

XPS spectra were recorded at different stages of the **CuP-1** catalyst lifetime. Both fresh calcined and H_2_-treated catalysts unambiguously contain Cu^2+^ species represented by a peak at 934.3 eV^128^ and a satellite peak at ∼944 eV typical for Cu^2+^ species^125^ (Figure 12A,B). No evident changes were observed during the hydrogen treatment step (1 h at 400 °C). The reduction of Cu^2+^ species was observed at the maximum catalytic activity (Figure 12C). Cu^2+^ species were reduced entirely in the spent catalyst to Cu^0^/Cu^+^ (peak at 932.8 eV). Unfortunately, Cu^0^ and Cu^+^ cannot be distinguished using the Cu 2p peak^129^ (Figure 12D), and the surface copper concentration is too low to observe Cu LMM signal (Auger electrons).^20^

The surface elemental composition for the different stages of catalyst lifetime, derived from XPS analyses, is presented in Table 5. The copper mass fraction increased according to XPS in the sample at the maximum catalytic activity (0.83 wt %) and then decreased for the spent catalyst (0.24 wt %). The amount of phosphorus on the surface of the spent catalyst significantly declined (Table 5). The surface phosphorus content decrease was also observed for **CuP-2**, **CuP-3**, and **CuP-P** when comparing fresh and spent catalysts (Table S5) and is obviously connected to the phosphorus leaching (confirmed by ICP-OES analysis and NMR spectroscopy).

**Table 5:** XPS Analysis of **CuP-1** at the Different Stages of the Catalyst Lifetime

**sample**	**Cuconc.** [wt %]	**Pconc.** [wt %]	**Cuconc.** [mol %]	**Pconc.** [mol %]	**Cu:P mol ratio**
Calcined	0.21	0.34	0.07	0.23	0.30
H_2_ treated	0.31	0.45	0.10	0.30	0.33
Top activity	0.83	0.50	0.27	0.33	0.83
Spent catalyst	0.24	0.05	0.07	0.03	2.3

**Figure 13:**
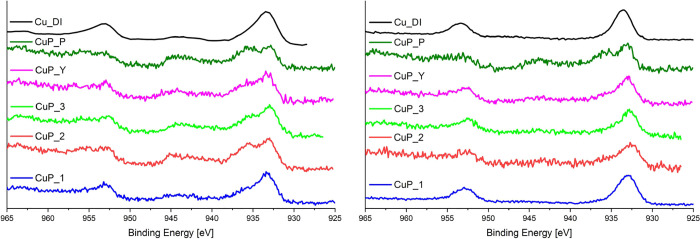
High-resolution XPS spectra (Cu 2p) recorded for all fresh calcined catalysts (left) and after TOS (right).

Figure 13 displays Cu 2p XPS spectra of **Cu-phosphate/SiO**_**2**_ samples (**CuP-1**, **CuP-2**, and **CuP-3**) in comparison with all benchmarks (**CuP-Y**, **CuP-P**, and **Cu-DI**); spectra of both fresh (calcined) and spent catalysts are shown. The presence of Cu^2+^ is detected in all calcined samples. The typical peak at ∼935 eV and satellite peak at ∼944 eV confirmed the presence of oxidized copper.^128^ The surface copper in **Cu-phosphate/SiO**_**2**_ samples was virtually reduced according to XPS after catalytic experiments, analogously to **Cu-DI**: the satellite peak at ∼944 eV diminished. An intense peak at ∼933 eV represents Cu^0^/Cu^+^ species which cannot be distinguished from each other.^129^ On the contrary, the **CuP-Y** and **CuP-P** benchmark samples after TOS still presented a clearly observable satellite peak confirming the presence of some oxidized Cu^2+^ species.

C 1s, O 1s, P 2p, and Si 2p XPS spectra of **Cu-phosphate/SiO**_**2**_ samples (**CuP-1**, **CuP-2**, and **CuP-3**) and all benchmarks (**CuP-Y**, **CuP-P**, and **Cu-DI**) are presented in Figure S18 (fresh and calcined) and Figure S19 (spent). The C 1s spectra show an expected presence of adventitious carbon, O 1s spectra present mostly oxygen in silica,^137^ binding energies observed in P 2p spectra agree well with the presence of phosphate species,^130^ and Si 2p spectra corroborate the nature of samples based on silica.^131,132^ No changes to the chemical or oxidation state were observed upon catalytic reaction, only phosphorus content decreased, as already discussed, and C content increased, probably due to the coke formation (see discussion below; Table S5).

Based on the results of XRD, ICP-OES, STEM-EDS, and XPS analyses described above, we propose a hypothesis regarding the possible activation and deactivation mechanisms in the **Cu-phosphate/SiO**_**2**_ catalysts. First, Cu^2+^ species in the **Cu-phosphate/SiO**_**2**_ catalysts and phosphorus-containing benchmark samples were not completely reduced (XPS) after H_2_ treatment and, therefore, exhibited a low activity. Cu reduction before ethanol dehydrogenation is a common pretreatment step and leads to catalyst activation.^16,133^ A possible explanation for the ineffective H_2_ treatment of the **Cu-phosphate/SiO**_**2**_ catalysts could be the presence of the Cu-phosphate phase with intimate Cu and P mixing, as suggested by STEM-EDS analyses (see Synthesis and Characterization of Cu-Phosphate/SiO2 Catalysts section). Metal phosphates are generally much more resistant to reduction than corresponding metal oxides.^134^

**Figure 14:**
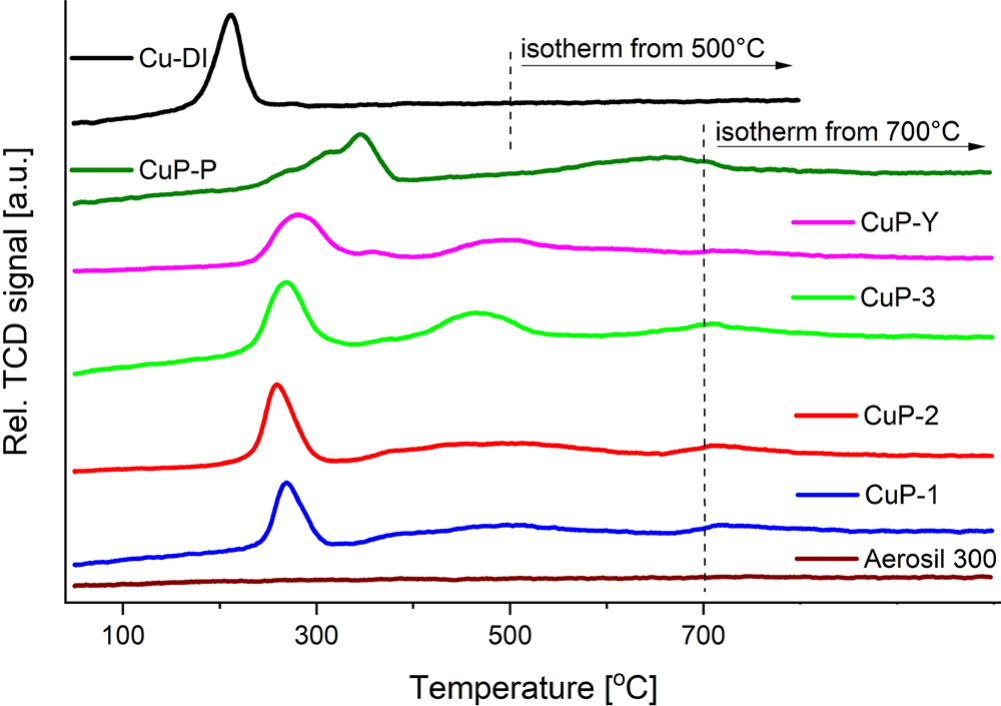
H_2_-TPR analysis of **Cu-phosphate/SiO**_**2**_ and the benchmark catalysts. The isothermal part of the measurements of the TCD signal was conducted at 700 °C for all phosphorus-containing samples and at 500 °C for the **Cu-DI** sample.

H_2_-TPR analyses support the first part of our hypothesis (Figure 14). Obviously, only the **Cu-DI** benchmark catalyst was fully reduced up to 400 °C, while **Cu-phosphate/SiO**_**2**_ catalysts were only partly reduced at this temperature. As a result, not all Cu species could participate in the catalytic reaction. The maximum of the main peak was observed at a temperature of 211 °C for the **Cu-DI** benchmark catalyst. This peak was shifted to higher temperatures in the case of **Cu-phosphate/SiO**_**2**_ catalysts (Figure 14; Table S6), similar to other Cu/P-SiO_2_ catalysts.^135^ Also, other peaks were found at higher temperatures (>400 °C; Figure 14; Table S6), and their shapes and positions were different for each individual sample. In the case of both phosphorus-containing benchmark catalysts (**CuP-Y** and **CuP-P**), the first main peak was broader; moreover, the most different shape of this peak was observed for the **CuP-P** benchmark catalyst (Figure 14; Table S6). The Cu concentrations calculated from the H_2_ consumptions during the H_2_-TPR analyses range from 1.7 to 2.6 wt % (Table S6) and are thus comparable to the Cu loadings estimated by ICP-OES analyses (Table 1).

The activated **CuP-1** catalyst (i.e., at the top of catalytic activity) exhibited a higher Cu:P ratio both in bulk and the surface layer (ICP-OES, Table 4; XPS, Table 5), smaller particle size (STEM; Figure 10), and a higher Cu surface content (XPS; Table 5) than the nonactive (freshly calcined) sample. Also, the Cu^2+^ species were mostly reduced to Cu^0^/Cu^+^ during time-on-stream (TOS) according to XPS analyses (Figure 12). Apparently, phosphorus leaching from the **Cu-phosphate/SiO**_**2**_ catalysts during TOS enabled Cu reduction. These processes could be simultaneous. Ethanol and H_2_ (originating in ethanol dehydrogenation) can be put forward as possible reducing agents. As already discussed, Cu reduction is necessary for its activation in the ethanol dehydrogenation reaction.^136^ The decrease in particle size and increase in the Cu surface content may also have a beneficial effect on the catalytic activity as well. It correlates well with the observed increase in catalytic activity during the first 1.6–8.3 h of catalytic reaction in **CuP-1**–**3**.

Finally, the **Cu-phosphate/SiO**_**2**_ samples started to deactivate after a period of high catalytic activity. Two deactivation mechanisms are primarily discussed in the literature: sintering of Cu particles and coking.^16−18,122^ Cu sintering has not been observed in our case: the samples remained XRD amorphous (Figure 11), and no large particles were observed by STEM-EDS (Figure 10). Therefore, we focused our attention on possible coking and analyzed the carbon content by XPS. The XPS analysis of the catalyst surface suggests that there is no significant coking, as the carbon content at the maximum of catalytic activity (3.49 wt %) is virtually the same as in that of the spent catalyst after 50 h of TOS (3.51 wt %), as shown in Table 6. The other spent phosphorus-containing catalysts exhibited a similar surface C content (2.93–3.56 wt %), while **Cu-DI** showed a somewhat higher surface C concentration (4.33 wt %; Table S5). The presence of coking is usually clearly observed after such extensive TOS.^20^ Notably, our findings agree with previous studies, which suggest that adding phosphorus to zeolite catalysts can prevent coking.^105,106^ Based on the results, an alternative deactivation mechanism can be suggested. The STEM-EDS micrographs of spent catalyst demonstrate a significant decrease in particle size (from 3.4 to 2.3 nm on average; ∼32%). At the same time, the Cu surface content significantly decreased (XPS; Table 5). Therefore, we hypothesize that Cu might diffuse into the SiO_2_ support and become inaccessible and, in turn, inactive. The stable Cu content in **CuP-1** at the different stages of catalyst lifetime (∼1.8 wt %; ICP-OES; Table 4) excludes the catalyst deactivation by the copper leaching. The Cu diffusion in silica-based catalysts has already been observed at the temperatures below *T*_Tamman_.^21^

**Table 6:** Study of Coking by XPS Analysis of **CuP-1** at the Different Stages of Catalyst Life

**sample**	**carbon content** [wt %]
Calcined	2.24
H_2-_ treated	3.74
Top activity	3.49
Spent catalyst	3.51

## Conclusions

In this study, three new Cu-phosphate-based catalysts were prepared using three newly synthesized well-soluble Cu(II) phosphinate complexes of different nuclearities: a polymeric (**1**), a hexanuclear (**2**), and a trinuclear (**3**) complex. The structures of complexes **1**–**3** were fully characterized and described in detail. Impregnation of the molecular precursors **1**–**3** on silica provided **Cu-phosphate/SiO**_**2**_ materials with homogeneously dispersed nanoparticles (2.1–3.4 nm) and narrow particle size distribution. The polymeric complex (**1**) provided larger particles with a broader distribution than the hexanuclar (**2**) and trinuclear (**3**) complexes. The particles consisted of both Cu and P according to STEM-EDS analyses. The **Cu-phosphate/SiO**_**2**_ materials were evaluated as catalysts in nonoxidative ethanol dehydrogenation and compared with the conventional benchmark Cu-based catalyst prepared by dry impregnation (**Cu-DI**), as well as Cu-based catalysts containing phosphorus (**CuP-Y** and **CuP-P**). Our results demonstrate a distinctively different catalytic behavior of the **Cu-phosphate/SiO**_**2**_ materials compared to the conventional Cu-based catalysts (**Cu-DI**). These materials also exhibited significantly higher activity when compared to those containing phosphorus (**CuP-Y** and **CuP-P**), but only comparable activity and lower stability in comparison to the phosphorus-free **Cu-DI** benchmark. By investigating the catalyst (**CuP-1**) life cycle, we uncovered the changes in the material influencing the catalytic properties. The increase of catalytic activity during TOS was related to the decrease of phosphorus content in both bulk and the surface layer and to the copper reduction. The maximum ethanol conversion was reached during 1.6–8 h of TOS, namely, ca. 73% for **CuP-1**, ca. 40% for **CuP-2**, and ca. 60% for **CuP-3**, while selectivity to acetaldehyde remained over 98% at WHSV = 2.37 h^–1^ for all catalysts. However, a steady decrease in catalytic activity was observed after the catalysts reached their maximum performance. The deactivation process was suggested to be related to Cu diffusion: the surface Cu concentration decreased together with the average particle size. The comparison of stability with TOS suggests that different strategies need to be explored to improve the long-term stability of Cu-based catalysts. Overall, our study demonstrates a new approach to P-doped Cu-based catalysts with intimate Cu and P mixing and sheds light on their peculiar behavior in nonoxidative ethanol dehydrogenation.

## Supporting Information Available

Synthesis of the ligands and their characterization (ICP-OES, IR, and NMR); synthesis of copper phosphinate complexes and their characterization (ICP-OES, IR, and ESI-MS), crystallographic parameters, selected bond lengths, CShM analysis of the polyhedra of the prepared complexes, details of catalysts preparation (masses of copper phosphinates and support), XPS surface measurement of Cu, P, and C, and H_2_-TPR results, ESI-MS, TG, PXRD, IR analyses of the prepared and calcined complexes, and adsorption isotherms of prepared catalysts and support; STEM-EDS analysis of benchmark **CuP-Y** and **CuP-P** samples after catalysis, and NMR spectra of obtained TEP, and XPS surface measurement of C, O, P, and Si on freshly calcined and spent catalysts (PDF)

## Abbreviations

CShM: continuous shape measures
DI: dry impregnation
EDS: energy dispersive spectroscopy
FID: flame ionization detector
IR: infrared spectroscopy
FTIR: Fourier transformation infrared spectroscopy
ATR: attenuated total reflectance
ICP-OES: inductively coupled plasma optical emission spectroscopy
STEM-EDS: scanning transmission electron microscopy with energy dispersive spectroscopy
TG: thermogravimetry
TOS: time-on-stream
XPS: X-ray photoelectron spectroscopy
