# Supplementary material for: Copper Phosphinate Complexes as Molecular Precursors for Ethanol Dehydrogenation Catalysts
Source: Inorg Chem. 2023 Nov 30;62(49):19871–86. doi: 10.1021/acs.inorgchem.3c01678 (PMC10716910; doi:10.1021/acs.inorgchem.3c01678)
Supplement: Supplementary file 1 — ic3c01678_si_001.pdf [file ic3c01678_si_001.pdf]

Supplementary materials for:

Copper Phosphinate Complexes as Molecular Precursors for Ethanol Dehydrogenation Catalysts

Tomas Pokorny[#a], Iaroslav Doroshenko[#a], Petr Machac[a], Lucie Simonikova[a], Miroslava Bittova[a], Zdenek Moravec[a], Katerina Karaskova[b], David Skoda[c], Jiri Pinkas[a], and Ales Styskalik[a]*

[a]Department of Chemistry, Faculty of Science, Masaryk University, Kotlarska 2, CZ-61137 Brno, Czech Republic

[b]Institute of Environmental Technology, CEET, VSB-TUO, CZ-70800 Ostrava, Czech Republic

[c]Centre of Polymer Systems, Tomas Bata University in Zlin, Tr. Tomase Bati 5678, 76001 Zlin, Czech Republic

* Corresponding author. Tel.: +420549498702
E-mail: styskalik@chemi.muni.cz (A. Styskalik).

[#] Tomas Pokorny and Iaroslav Doroshenko contributed equally to this work and should be considered co-first authors.

Ligands synthesis

(2-aminopropan-2-yl)phenylphosphinic acid (**HAIPPA**)

HAIPPA was synthesized according to the literature method.[1] Commercially available benzyl carbamate, dichlorophenylphosphine, acetone, and acetic acid were used. The main product was hydrolyzed by hydrochloric acid, and the formed hydrochloride was transformed into free aminophosphonic acid by adding propylene oxide to its methanolic solution. Phenylphosphonic acid was observed as the main impurity. The boiling of the impure product with propan-2-ol, further filtration of the obtained slurry, and washing of the filtered precipitate with propan-2-ol allowed for the separation of the phenylphosphonic acid from the main product.

Elemental analysis (ICP-OES, calculated for $C_9H_{14}NO_2P$/found): P 15,55/15,31%.

IR (cm^{-1}): ν 3126 vw, 3072 vw, 3055 vw, 2990 vw, 2973 vw, 2916 vw, 2823 vw, 2758 vw, 2716 vw, 2658 vw, 2606 vw, 2556 vw, 2470 vw, 2294 vw, 2163 vw, 1651 vw, 1606 vw, 1552 m, 1482 vw, 1466 vw, 1453 vw, 1436 vw, 1393 vw, 1372 vw, 1313 vw, 1267 vw, 1159 vs, 1127 vs, 1071 w, 1049 vs, 1025 m, 997 w, 930 vw, 836 w, 759 vw, 748 w, 713 m, 702 s, 663 vw, 630 s, 544 s, 530 s, 504 vw, 457 w, 429 w, 415 w.

^{1}H NMR (300.1 MHz, D_2O, ppm): δ 1.37 (d, *J* = 12.1 Hz, 6H –CH_3), 4.75 (s, D_2O), 7.59 (m, 3H, Ph), 7.76 (m, 2H, Ph).

^{31}P {^{1}H} NMR (121.5 MHz, D_2O, ppm): δ 27.81.

Sodium (2-{[(E)-(2-hydroxyphenyl)methylidene]amino}propan-2-yl)phenylphosphinate (**NaHSAAP**)

Salicylaldehyde (1.520 g, 12.45 mmol) was added to the solution of HAIPPA (2.480 g, 12.45 mmol) and NaOH (0.498 g, 12,5 mmol) in MeOH (100 cm^3). The color of the solution immediately became canary-yellow. The solution was kept under reflux overnight. The volume of the solution was then reduced to 5–10 cm^3 using a rotary evaporator, and the product was precipitated by adding a large amount of acetonitrile. The yellow product was then filtered off, washed with acetonitrile, dried in the open air, and then for 48 h under vacuum (80 ^{o}C, 2.5 10^{-3} mbar), resulting in 3.646 g of dried NaHSAAP (yield of 90.0% based on P, $M(C_{16}H_{17}NNaO_3P)$ = 325.28 g mol^{-1}).

Elemental analysis (ICP-OES, calculated for $C_{16}H_{17}NNaO_3P$/found): Na 7.07/7.31; P 9.52/10.28.

IR (cm^{-1}): ν 3077 vw, 3051 vw, 3012 vw, 2983 vw, 2971 vw, 2866 vw, 1622 m, 1526 w, 1505 w, 1470 vw, 1435 vw, 1384 vw, 1384 vw, 1316 vw, 1249 vw, 1197 vs, 1143 m, 1129 m, 1055 s, 1026 w, 999 vw, 969 vw, 929 vw, 899 vw, 860 vw, 837 vw, 779 vw, 761 w, 749 w, 737 m, 718 s, 698 s, 664 w, 613 w, 557 s, 546 m, 520 w, 469 vw, 446 w.

^{1}H NMR (300.1 MHz, CD_3OD, ppm): δ 1.48 (d, *J* = 12.4 Hz, 6H –CH_3), 3.31 (m, CD_3OD), 4.81 (s, H_2O), 6.73 (m, 2H Ar), 7.32 (m, 5H Ar), 7.72 (m, 2H Ar), 8.25 (d, *J* = 3.2 Hz, 1H –CH=).

^{31}P {^{1}H} NMR (121.5 MHz, CD_3OD, ppm): δ 29.04.

Sodium (2-{[(E)-(5-bromo-2-hydroxyphenyl)methylidene]amino}propan-2-yl) phenylphosphinate (**NaHBSAAP**)

2-bromo-5-hydroxybenzaldehyde (1.456 g, 7.250 mmol) was added to the stirred solution of HAIPPA (0.962 g, 4.83 mmol) and NaOH (0.194 g, 4.83 mmol) in 100 cm^3 of MeOH. The solution color immediately changed into canary-yellow. The formed solution was kept under reflux overnight. Then, the volume of the solution was reduced to 15–30 cm^3 using a rotary evaporator. A yellow precipitate was formed after adding a large amount of acetonitrile. The yellow product was then filtered off, washed with acetonitrile, dried in the open air, and then for 48 h under vacuum (80 °C, 2.5 10^{-3} mbar), resulting in 1.356 g of NaHBSAAP (yield of 69.5% based on P, $M(C_{16}H_{16}BrNNaO_3P)$ = 404.17 g mol^{-1}).

Elemental analysis (ICP-OES, calculated for $C_{16}H_{16}BrNNaO_3P$/found): Na 5.69/6.02; P 7.66/6.79%.

IR (cm^{-1}): ν 3057 vw, 2985 vw, 2973 vw, 1631 w, 1561 vw, 1511 w, 1457 vw, 1439 vw, 1386 vw, 1366 vw, 1331 vw, 1315 vw, 1239 vw, 1196 vs, 1144 w, 1130 m, 1055 m, 1027 w, 1001 vw, 963 vw, 953 vw, 930 vw, 907 vw, 888 vw, 827 m, 781 vw, 753 m, 719 s, 701 m, 678 w, 631 w, 620 w, 560 vs, 550 m, 536 w, 522 vw, 482 vw, 469 vw, 448 w, 433 w.

^{1}H NMR (300.1 MHz, CD_3OD, ppm): δ 1.47 (d, *J* = 12.3 Hz, 6H, $-CH_3$), 3.31 (m, CD_3OD), 4.86 (s, H_2O), 6.70 (d, *J* = 9.0 Hz, 1H Ar), 7.36 (m, 5H), 7.70 (m, 2H Ar), 8.21 (d, *J* = 3.1 Hz, 1H –CH=).

^{31}P {^{1}H} NMR (121.5 MHz, CD_3OD, ppm): δ 28.72.

Sodium (2-{[(E)-(2-hydroxynaphthalen-1-yl)methylidene]amino}propan-2-yl) phenylphosphinate (**NaHNAAP**)

2-hydroxy-1-naphthaldehyde (1.613 g, 9.37 mmol) was added to the stirred solution of HAIPPA (1.886 g, 9.32 mmol) and NaOH (1.606 g, 9.32 mmol) in 100 cm^3 of MeOH. The solution color immediately changed into canary-yellow. The formed solution was kept under reflux overnight. Then, the volume of the solution was reduced to 15–30 cm^3 using a rotary evaporator. A yellow precipitate was formed after adding a large amount of acetonitrile. The yellow product was then filtered off, washed with acetonitrile, dried in open air, and then for 48 h under vacuum (80 °C, 2.5 10^{-3} mbar), resulting in 3.312 g of NaHNAAP (yield of 94.2% based on P, $M(C_{20}H_{19}NNaO_3P)$ = 375.33 g mol^{-1}).

Elemental analysis (ICP-OES, calculated for $C_{20}H_{19}NNaO_3P$/found): Na 6.13/6.19; P 8.25/8.09%.

IR (cm^{-1}): ν 3325 vw, 3054 vw, 3025 vw, 2981 vw, 2967 vw, 2930 vw, 2866 vw, 1620 vs, 1544 m, 1521 m, 1494 w, 1470 vw, 1447 vw, 1436 w, 1395 vw, 1384 vw, 1365 w, 1345 m, 1193 vs, 1126 s, 1043 vs, 1025 m, 1000 w, 938 vw, 856 vw, 834 m, 746 s, 714 vs, 699 s, 661 w, 627 m, 561 s, 536 vs, 511 m, 466 w, 442 w, 410 vw.

^{1}H NMR (300.1 MHz, CD_3OD, ppm): δ 1.58 (d, *J* = 11.9 Hz, 6H $-CH_3$), 6.74 (d, *J* = 9.5 Hz, 1H Ar), 7.19 (t, *J* = 14.9 Hz, 1H Ar), 7.36 (m, *J* = 37.0 Hz, 4H Ar), 7.58 (d, *J* = 7.8 Hz, 1H Ar), 7.72 (m, *J* = 19.0 Hz, 3H Ar), 7.88 (d, *J* = 8.3 Hz, 1H Ar), 8.82 (s, 1H –CH=).

^{31}P {^{1}H} NMR (121.5 MHz, CD_3OD, ppm): δ 27.93.

Synthesis of the complexes – catalyst precursors

{Cu(SAAP)}$_n$ (**1**)

$Cu(NO_3)_2·2.5H_2O$ (0.116 g, 0.500 mmol) was added to the vigorously stirred solution of NaHSAAP (0.169 g, 0.520 mmol) and NaOH (0.500 mmol) in 20 cm^3 of MeOH. The formed

emerald-green solution was evaporated to dryness. The dry residue was suspended in tetrahydrofuran and filtered to separate the soluble complex from undissolved $NaNO_3$. The solution was then dried, and the dried residue was dissolved in CH_3CN (40 cm^3) and left to stand for crystallization. One week later, small, needle-like crystals were obtained. The crystals were filtered, washed with CH_3CN, and dried in open air leading to 0.153 g of $\{Cu(SAAP)\}_n$ (yield of 83.9% based on P, $M(C_{16}H_{16}CuNO_3P)$ = 364.82 g mol^{-1}). Single crystals suitable for the single-crystal X-ray diffraction analysis were obtained by the vapor diffusion of acetone to the methanol solution of **1**.

Elemental analysis (ICP-OES, calculated for $C_{16}H_{16}CuNO_3P$/found): Cu 17.4/18.5; P 8.49/8.57%.

IR (cm^{-1}): ν 3072 vw, 3049 vw, 3022 vw, 2968 vw, 2958 vw, 2922 vw, 2858 vw, 1625 s, 1598 m, 1553 w, 1474 m, 1442 m, 1383 w, 1373 vw, 1358 vw, 1313 vw, 1283 s, 1258 vw, 1207 s, 1194 s, 1153 w, 1146 w, 1122 s, 1070 vw, 1042 vw, 1032 w, 1008 s, 995 s, 978 w, 967 w, 934 vw, 904 w, 855 w, 847 w, 795 w, 758 s, 748 m, 720 vs, 693 s, 638 vw, 604 s, 591 m, 559 vs, 522 m, 479 m, 441 m, 430 m.

ESI-MS (positive ion mode, MeOH, 50 V): *m/z* 1095.06 $[Cu_3(SAAP)_3 + H]^+$, 3.01%; 1117.04 $[Cu_3(SAAP)_3 + Na]^+$, 8.97%; 1481.05 $[Cu_4(SAAP)_4 + Na]^+$, 100%; 1847.07 $[Cu_5(SAAP)_5 + Na]^+$, 64.9%.

ESI-MS (positive ion mode, MeOH, 100 V): *m/z* 729.04 $[Cu_2(SAAP)_2 + H]^+$, 22.9%; 1095.06 $[Cu_3(SAAP)_3 + H]^+$, 100%; 1355.04 $[Cu_4(SAAP)_4(AIPPA)]^+$, 32.9%; 1459.07 $[Cu_4(SAAP)_4 + H]^+$, 48.5%; 1481.05 $[Cu_4(SAAP)_4 + Na]^+$, 27.7%; 1522.99 $[Cu_4(SAAP)_4 + Cu]^+$, 7.78%; 1721.06 $[Cu_5(SAAP)_5(AIPPA)]^+$, 8.14%; 1847.07 $[Cu_5(SAAP)_5 + Na]^+$, 16.92%.

ESI-MS (negative ion mode, MeOH, 100 V): *m/z* 425.99 $[Cu(SAAP) + NO_3]^-$, 100%.

$[Cu_6(BSAAP)_6]$ (**2**)

The solution of $Cu(NO_3)_2 \cdot 2.5H_2O$ (0.116 g, 0.500 mmol) in 10 cm^3 of MeOH was added to the vigorously stirred solution of NaHBSAAP (0.202 g, 0.500 mmol) and NaOH (0.500 mmol) in 10 cm^3 of MeOH. The obtained emerald-green solution was left to stand for one day. Then, the solvent was evaporated to dryness. THF (10 cm^3) was added to the dried residue, and the formed solution was filtered to separate the soluble complex from undissolved $NaNO_3$. The solution was evaporated to dryness, and the dried glass-like residue was dissolved in 20 cm^3 of CH_3CN. The solution was left to stand for one week for crystallization. The formed crystalline precipitate was then filtered, washed with CH_3CN, and dried in open air leading to 0.155 g of $[Cu_6(BSAAP)_6]$ (yield of 69.9 % based on P, $M(C_{96}H_{90}Br_6Cu_6N_6O_{18}P_6)$ = 2662.31 g mol^{-1}). Single-crystals suitable for the single-crystal X-ray diffraction analysis were obtained by the complex crystallization from the CH_3CN solution with a low concentration.

Elemental analysis (ICP-OES, calculated for $C_{96}H_{90}Br_6Cu_6N_6O_{18}P_6$/found): Cu 14.3/14.3; P 6.98/6.85%.

IR (cm^{-1}): ν 3054 vw, 2963 vw, 1622 m, 1593 w, 1523 w, 1464 m, 1436 w, 1416 vw, 1383 m, 1314 vw, 1279 m, 1204 m, 1182 s, 1156 s, 1145 s, 1122 vs, 1069 vw, 1037 m, 1021 m, 1005 m, 933 s, 940 vw, 916 vw, 855 vw, 832 w, 818 w, 801 w, 749 w, 729 vs, 695 s, 647 m, 603 w, 588 m, 562 vs, 543 s, 533 s, 505 w, 481 w, 442 m.

ESI-MS (positive ion mode, MeOH. 50 V): *m/z* 1330.79 [$Cu_3(BSAAP)_3$ + H]$^+$, 47.5%; 1774.71 [$Cu_4(BSAAP)_4$ + H]$^+$, 26.7%; 1796.70 [$Cu_4(BSAAP)_4$ + Na]$^+$, 100%; 2240.62 [$Cu_5(BSAAP)_5$ + Na]$^+$, 59.9%; 2662.56 [$Cu_6(BSAAP)_6$ + H]$^+$, 3.94%.

ESI-MS (positive ion mode, MeOH. 100 V): *m/z* 888.85 [$Cu_2(BSAAP)_2$ + H]$^+$, 1.56%; 910.84 [$Cu_2(BSAAP)_2$ + Na]$^+$, 2.58%; 1330.79 [$Cu_3(BSAAP)_3$ + H]$^+$, 86.85%; 1352.77 [$Cu_3(BSAAP)_3$ + Na]$^+$, 4.54%; 1592.77 [$Cu_4(BSAAP)_3$(AIPPA)]$^+$, 3.88%; 1774.71 [$Cu_4(BSAAP)_4$ + H]$^+$, 33.64%; 1796.70 [$Cu_4(BSAAP)_4$ + Na]$^+$, 100%; 2240.62 [$Cu_5(BSAAP)_5$ + Na]$^+$, 57.89%; 2662.56 [$Cu_6(BSAAP)_6$ + H]$^+$, 3.85%.

ESI-MS (negative ion mode, MeOH, 100 V): *m/z* 478.88 [Cu(BSAAP) + Cl]$^-$, 17.05%; 505.90 [Cu(BSAAP) + NO_3]$^-$, 100%; 918.84 [$Cu_2(BSAAP)_2$ + OMe]$^-$, 41.25%; 949.81 [$Cu_2(BSAAP)_2$ + NO_3]$^-$,12.78%; 1360.76 [$Cu_3(BSAAP)_3$ + OMe]$^-$, 8.58%.

[$Cu_3(NAAP)_3$] (**3**)

The solution of $Cu(NO_3)_2\cdot 2.5H_2O$ (0.144 g, 0.621 mmol) in 10 cm^3 of MeOH was added to the vigorously stirred solution of NaHNAAP (0.233 g, 0.621 mmol) and NaOH (0.621 mmol) in 10 cm^3 of MeOH. The obtained emerald-green solution was left to stand for one day. Then the solvent was evaporated to dryness. THF (10 cm^3) was added to the dried residue, and the formed solution was filtered to separate the soluble complex from undissolved $NaNO_3$. The solution was evaporated to dryness, and the dried glass-like residue was dissolved in 50 cm^3 of CH_3CN and then the solution volume was reduced to 5–10 cm^3 till the crystallization began. After one week, crystals were filtered, washed with a small amount of acetonitrile, and dried in open air leading to 0.211 g of [$Cu_3(NAAP)_3$] (yield of 81.9% based on P, $M(C_{60}H_{54}Cu_3N_3O_9P_3)$ = 1244.65 g mol^{-1}).

Elemental analysis (ICP-OES, calculated for $C_{60}H_{54}Cu_3N_3O_9P_3$/found): Cu 15.3/15.7; P 7.47/7.32%.

IR (cm^{-1}): 3055 vw, 2985 vw, 2967 vw, 2928 vw, 1616 m, 1604 m, 1588 m, 1538 m, 1505 w, 1455 w, 1429 m, 1398 m, 1385 m, 1356 m, 1340 w, 1304 w, 1252 vw, 1208 w, 1189 w, 1161 s, 1144 m, 1125 s, 1099 w, 1090 w, 1071 vw, 1033 w, 1018 w, 994 m, 966 w, 949 m, 855 vw, 826 m, 771 w, 745 m, 722 s, 693 m, 678 w, 644 vw, 594 m, 566 vs, 543 s, 515 m, 504 w, 456 w, 450 w, 424 vw, 411 w.

ESI-MS (positive ion mode, MeOH, 100V, lower conc): *m/z* 851.05 [$Cu_2(NAAP)_2$ + Na]$^+$, 3.66%; 1245.10 [$Cu_3(NAAP)_3$ + H]$^+$, 100%; 1267.09 [$Cu_3(NAAP)_3$ + Na]$^+$, 7.29%.

ESI-MS (positive ion mode, MeOH, 100V, higher conc): *m/z* 851.06 [$Cu_2(NAAP)_2$ + Na]$^+$, 1.98%; 1245.11 [$Cu_3(NAAP)_3$ + H]$^+$, 18.65%; 1267.09 [$Cu_3(NAAP)_3$ + Na]$^+$, 4.29%; 1659.14 [$Cu_4(NAAP)_4$ + H]$^+$, 8.76%; 1681.12 [$Cu_4(NAAP)_4$ + Na]$^+$, 67.46%; 2097.16 [$Cu_5(NAAP)_5$ + Na]$^+$, 100%.

ESI-MS (negative ion mode, MeOH, 100V, higher conc): *m/z* 861.05 [$Cu_2(NAAP)_2$ + OMe]$^-$, 100%; 1275.07 [$Cu_3(NAAP)_3$ + OMe]$^-$, 11.10%.

Table S1. Selected crystallographic data and structure refinement parameters for the complexes **1**–**3.**

Parameters	**1**	**2**	**3**
Formula[a]	$C_{16}H_{16}CuNO_3P$	$C_{53}H_{51}Br_3Cu_3N_{5.5}O_9P_3$	$C_{62}H_{57}Cu_3N_4O_9P_3$
fw (g mol^{-1})	364.81	1432.25	1285.64
Cryst syst	monoclinic	triclinic	triclinic
Space group	$P2/c$	$P\bar{1}$	$P\bar{1}$
a (Å)	12.3074(3)	14.4933(2)	12.48312(19)
b (Å)	8.3718(2)	14.9422(2)	14.22030(19)
c (Å)	15.0596(3)	15.9203(2)	17.15529(13)
α (deg)	90	70.3367(14)	108.5138(10)
β (deg)	104.540(2)	72.2742(13)	90.5805(11)
γ (deg)	90	64.6503(14)	90.7658(12)
V (Å^3)	1501.97(6)	2881.48(8)	2887.11(6)
Z	4	2	2
T (K)	120(2)	120(2)	120(2)
δ_{calc} (g cm^{-3})	1.613	1.651	1.479
F(000)	748	1433	1322
μ(Mo-K$_\alpha$) (mm^{-1})	3.165	3.319	2.569
θ range of data collection (deg)	3.710–69.985	1.5532–30.9839	3.5313–78.1587
Measured reflections	6628	39550	87160
Unique reflections (R_{int})	2841 (0.0328)	10154 (0.0208)	10869 (0.0622)
no. of param	201	696	737
GOF on F^2 [b]	1.060	1.035	1.061
R_1 [$I > 2\sigma(I)$]	0.0246	0.0345	0.0472
wR_2 (all data)[c]	0.0717	0.0944	0.1282
$\Delta\rho_{max}$ (e Å^{-3})	0.329	1.707	1.175
$\Delta\rho_{min}$ (e Å^{-3})	-0.421	-0.568	-0.895
CCDC	2252549	2252550	2252551

[a] The molecular formulas and molecular weights corresponding to the data obtained from the single-crystal X-ray analysis were used in this table. These formulas could differ from the ones obtained by elemental analysis of dried samples.

[b] $$\mathrm{GOF} = \left(\frac{\Sigma\left[w\left(F_o^2-F_c^2\right)^2\right]}{\left(N_o-N_p\right)}\right)^{1/2}.$$

[c] $$R_1 = \frac{\Sigma\left||F_o|-|F_c|\right|}{\Sigma|F_o|};\ wR_2 = \left(\frac{\Sigma\left[w\left(F_o^2-F_c^2\right)^2\right]}{\Sigma\left[w\left(F_o^2\right)^2\right]}\right)^{1/2}$$ with

$$w^{-1} = \sigma^2(F_o^2) + (aP)^2 + bP;\ P = \frac{2F_c^2+\max F_o^2}{3}.$$

Table S2. Bond lengths (Å) in copper(II) polyhedra.

Complex	Central atom	Donor atom	Length [Å]
**1**	Cu1	O1	1.957(1)
		O2	2.184(1)
		O3	1.9393(9)
		O3	1.993(1)
		N1	1.989(1)
**2**	Cu1	O1_1	1.965(3)
		O3_1	1.895(3)
		N1_1	1.953(3)
		O2_2	1.952(2)
	Cu2	O1_2	1.974(3)
		O3_2	1.954(2)
		N1_2	1.972(2)
		O2_3	2.181(3)
		O3_3	1.981(2)
	Cu3	O1_3	1.925(3)
		O3_3	1.942(2)
		N1_3	1.978(2)
		O2_1	2.234(3)
		O3_2	2.023(2)
**3**	Cu1	O1_1	1.956(2)
		O3_1	1.890(2)
		N1_1	1.958(2)
		O2_2	1.946(1)
	Cu2	O1_2	1.938(1)
		O3_2	1.893(2)
		N1_2	1.942(2)
		O1_1	2.321(1)
		O1_3	1.996(1)
	Cu3	O1_3	1.981(2)
		O3_3	1.888(2)
		N1_3	1.945(2)
		O2_1	1.955(1)
		O3_2	2.395(1)

Table S3. The geometry analysis of Cu^{2+} polyhedra in the complexes **1**, **2**, and **3** by the SHAPE 2.1 program.[2–5]

Complex	Central atom	**PP-5**[a]	**vOC-5**[b]	**TBPY-5**[c]	**SPY-5**[d]	**JTBPY-5**[e]
**1**	Cu1	32.095	2.508	2.714	1.553	6.005
**2**	Cu2	31.843	1.127	4.416	0.726	7.026
	Cu3	30.979	1.945	3.581	1.319	6.132
**3**	Cu2	32.310	1.088	4.341	1.079	7.604
	Cu3	26.015	3.298	3.805	2.550	7.259
		**SP-4**[f]	**T-4**[g]	**SS-4**[h]	**vTBPY-4**[i]	-
**2**	Cu1	3.862	16.997	8.508	18.852	-
**3**	Cu1	2.506	19.764	9.645	21.624	-

[a] Pentagon; [b] Vacant octahedron C_{4v}; [c] Trigonal bipyramidal; [d] Spherical square pyramid; [e] Johnson trigonal bipyramid J12; [f] Square D_{4h}; [g] Tetrahedron; [h] Seesaw C_{2v}; [i] Vacant trigonal biyramid; [j] Hexagon D_{6h}; [k] Pentagonal pyramid C_{5v}; [l] Octahedron O_h; [m] Trigonal prism D_{3h}; [n] Johnson pentagonal pyramid J2 C_{5v}.

Table S4. Used masses of Cu phosphinate complexes for catalysts preparation

Sample	Cu phosphinate complex [g]	Aerosil 300 [g]	Nominal Cu content [wt%]
**CuP-1**	0.149	1.00	2.5
**CuP-2**	0.081	0.454	2.5
**CuP-3**	0.167	1.00	2.5
**CuP-3-TEP**	1.033	0.397	25[a]

[a] calculated with regard to phosphorus amount.

Table S5. XPS surface measurement of Cu, P, and C content in Cu-phosphate/SiO_2 samples and comparison with the benchmark catalysts.

Sample	Calcined				Spent			
	Cu [wt%]	P [wt%]	C [wt%]	Cu : P Ratio	Cu [wt%]	P [wt%]	C [wt%]	Cu : P Ratio
**CuP-1**	0.21	0.34	2.24	0.30	0.24	0.05	3.51	2.3
**CuP-2**	0.37	0.51	1.15	0.35	0.19	0.21	3.56	0.43
**CuP-3**	0.17	0.73	1.16	0.11	0.28	0.18	2.93	0.75
**CuP-Y**	0.34	0.45	0.71	0.37	0.31	0.62	3.07	0.24
**CuP-P**	0.34	0.72	1.52	0.23	0.34	0.32	2.96	0.52
**Cu-DI**	0.43	-	1.85	-	0.40	-	4.33	-

Table S6. H_2-TPR results.

Sample	T_{max} [°C]	H_2 consumption [mmol g^{-1}][a]	Cu [wt%][b]
**CuP-1**	269, 492, 701	0.29	1.9
**CuP-2**	259, 452, 702	0.35	2.2
**CuP-3**	269, 464, 703	0.41	2.6
**CuP-Y**	281, 357, 500, 702	0.28	1.8
**CuP-P**	345, 661	0.27	1.7
**Cu-DI**	211	0.41	2.6

[a] Consumption in temperature range 50–500 °C.

[b] Calculated from H_2 consumption.

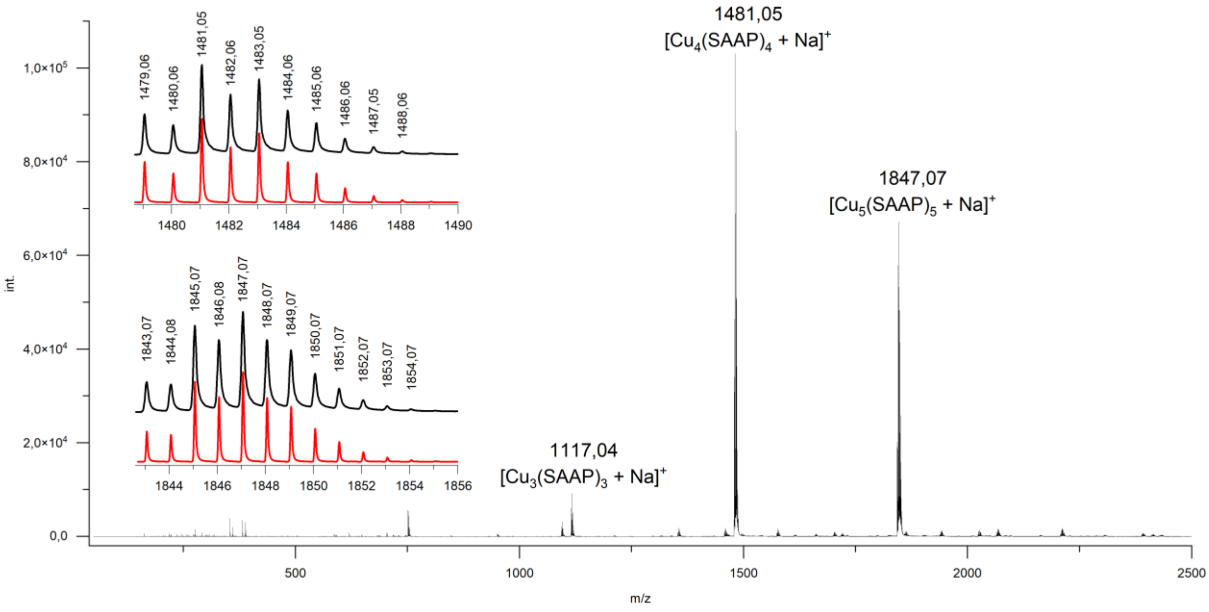

Figure S1. ESI-MS spectra of **1** in positive mode (fragmentor 50 V). Insets represent calculated (red) and experimental (black) isotopic patterns of the most intense peaks.

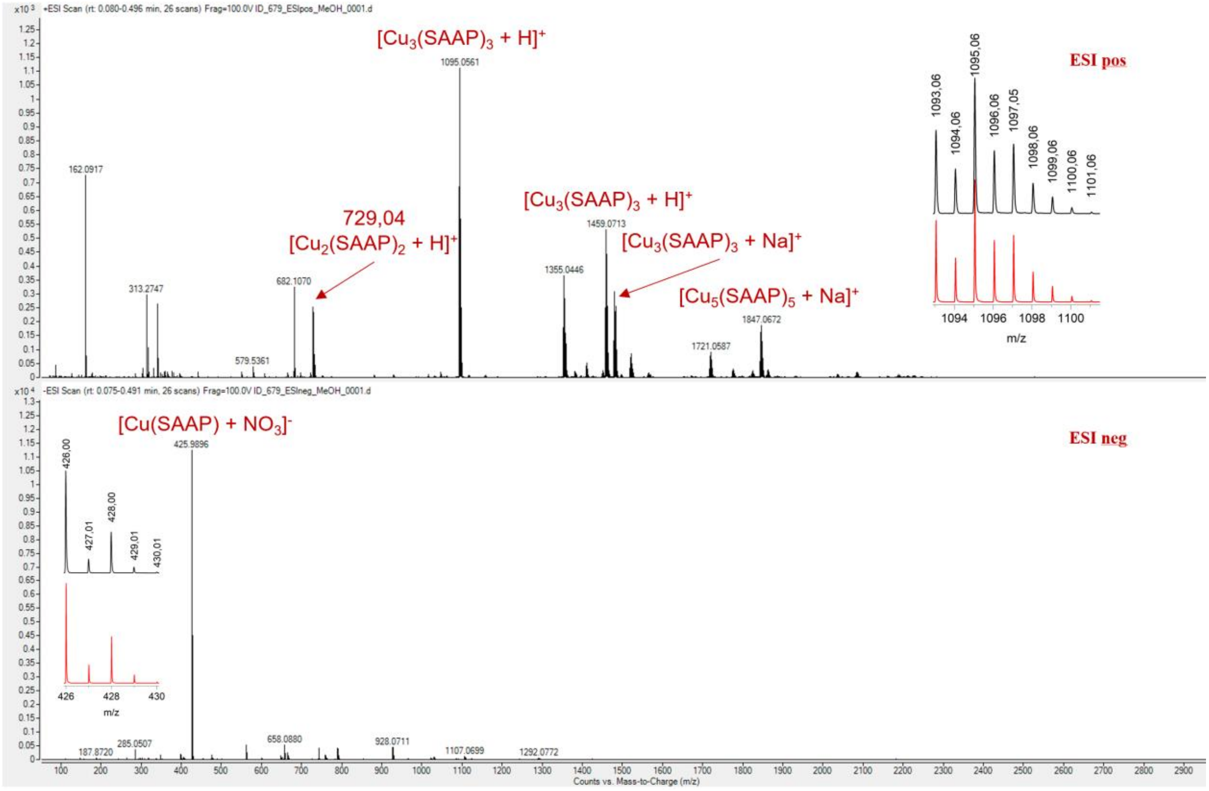

Figure S2. ESI-MS spectra of **1** in positive and negative modes (fragmentor 100 V). Insets represent calculated (red) and experimental (black) isotopic patterns of the most intense peaks.

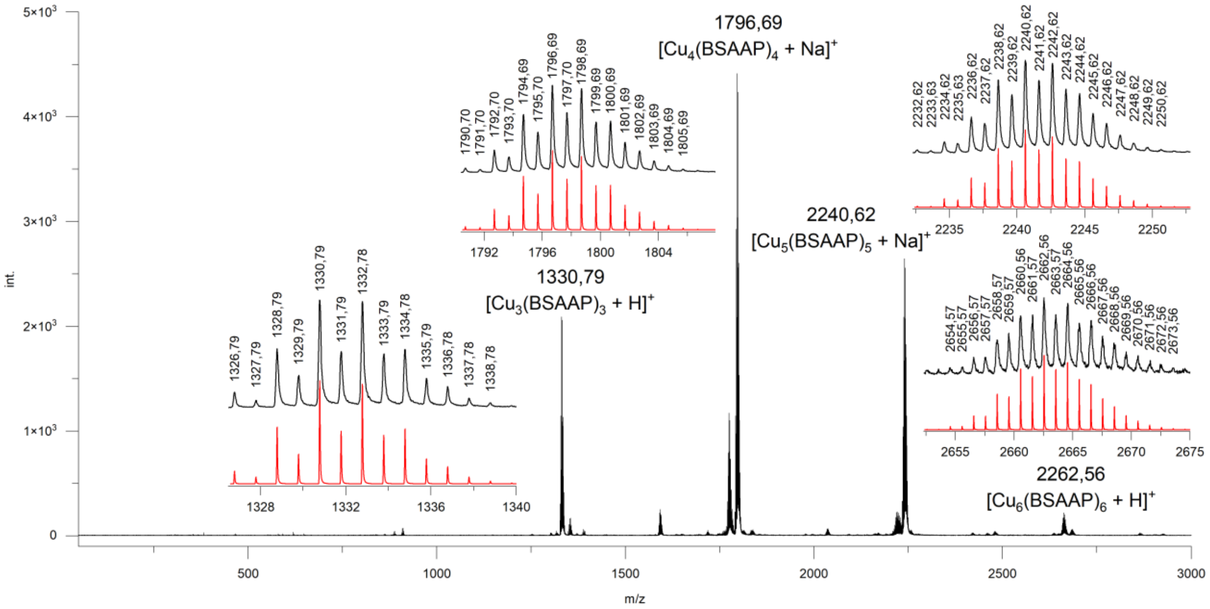

Figure S3. ESI-MS spectra of **2** in positive mode (fragmentor 50 V). Insets represent calculated (red) and experimental (black) isotopic patterns of the most intense and molecular peaks.

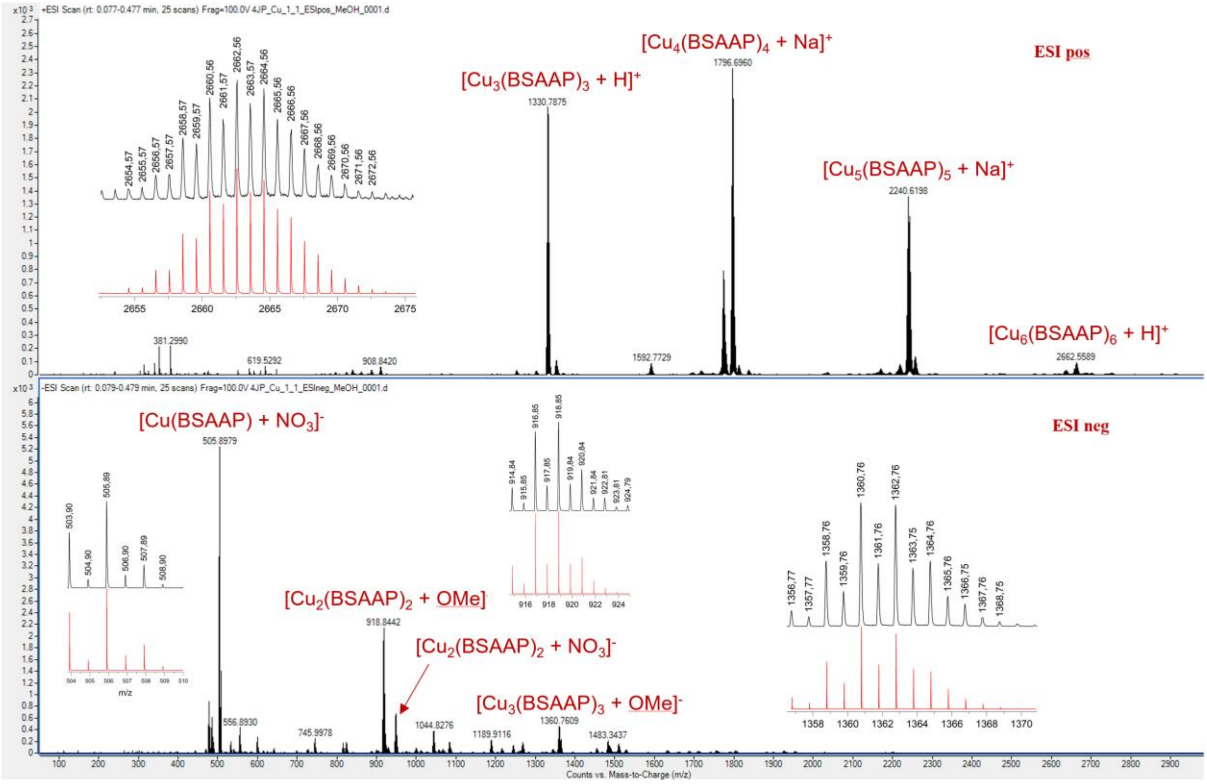

Figure S4. ESI-MS spectra of **2** in positive and negative modes (fragmentor 100 V). Insets represent calculated (red) and experimental (black) isotopic patterns of the most intense and molecular peaks.

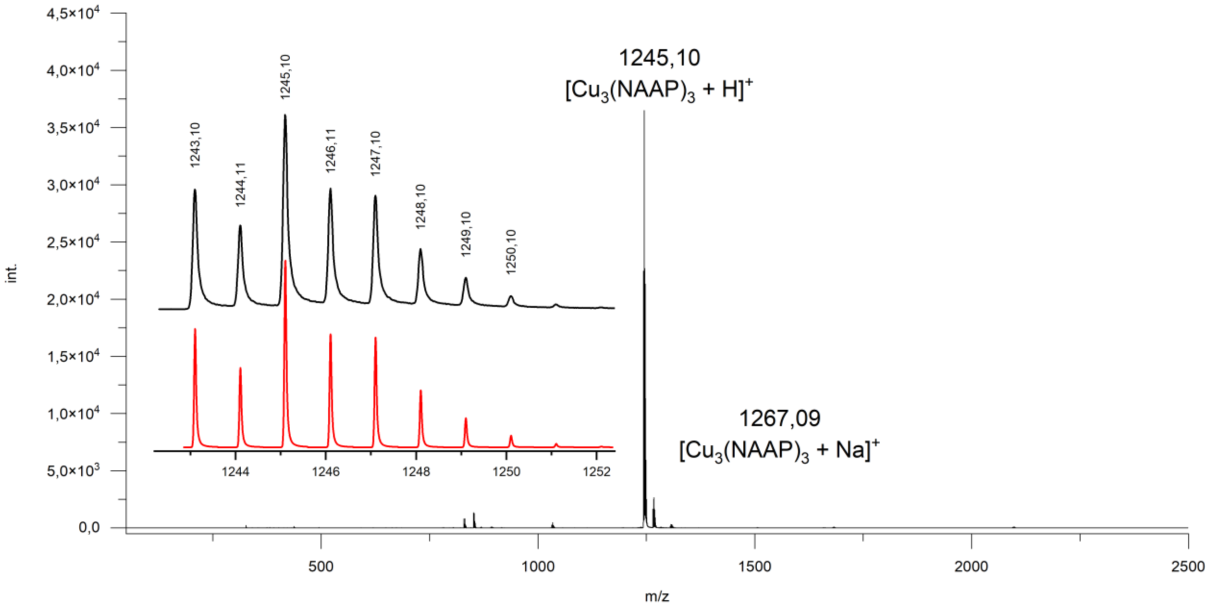

Figure S5. ESI-MS spectra of **3** in positive mode (fragmentor 100 V, lower concentration). Inset represents calculated (red) and experimental (black) isotopic patterns of the most intense molecular peak.

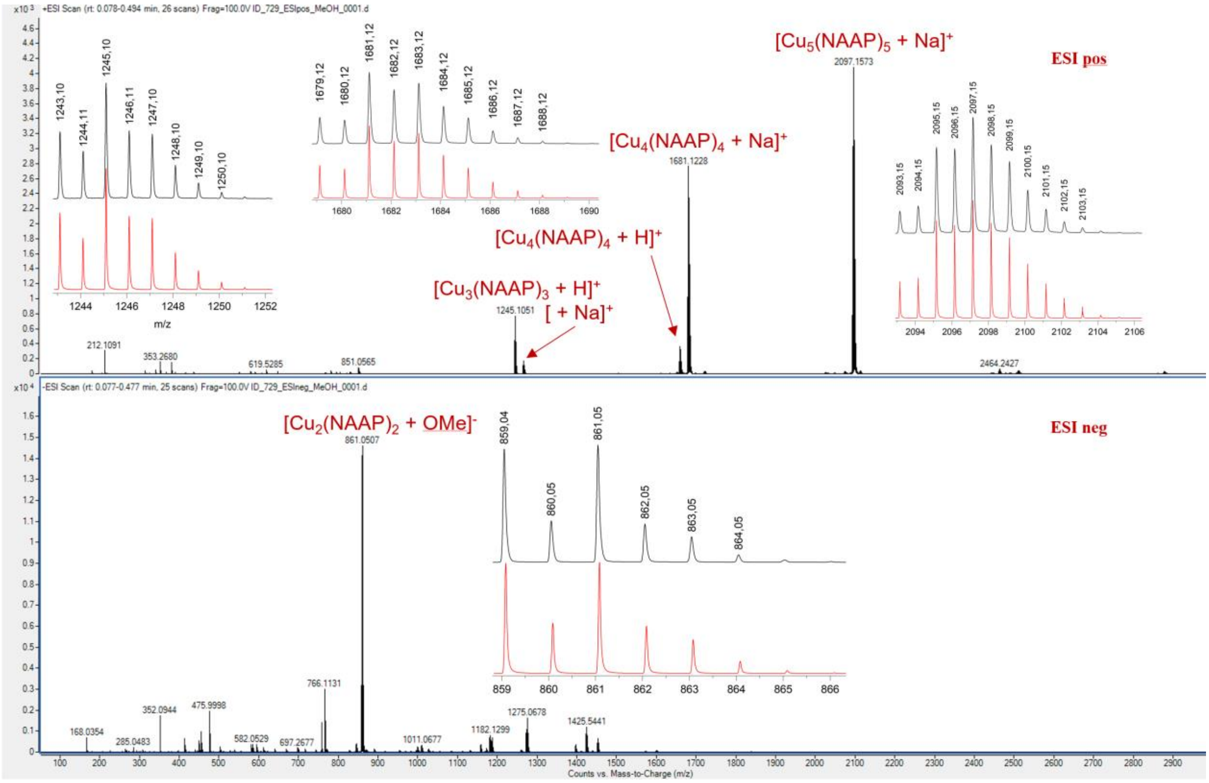

Figure S6. ESI-MS spectra of **3** in positive and negative modes(fragmentor 100 V, higher concentration). Insets represent calculated (red) and experimental (black) isotopic patterns of the most intense and molecular peaks.

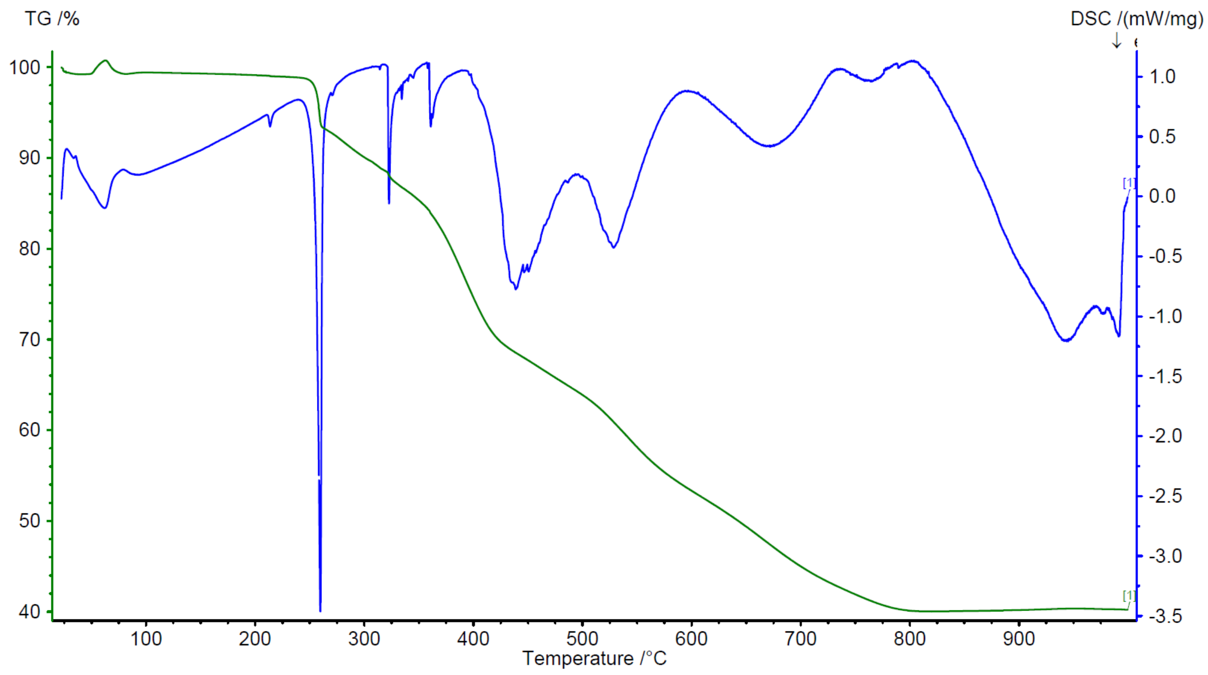

Figure S7. TG and DSC curves of **1**.

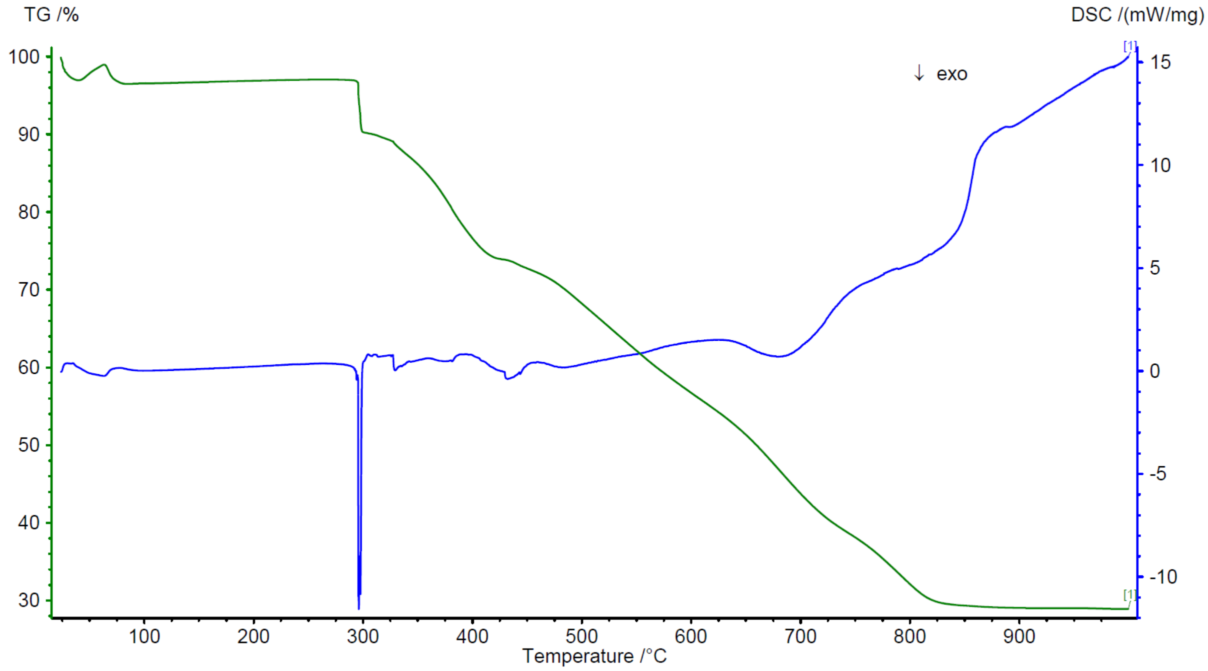

Figure S8. TG and DSC curves of **2**.

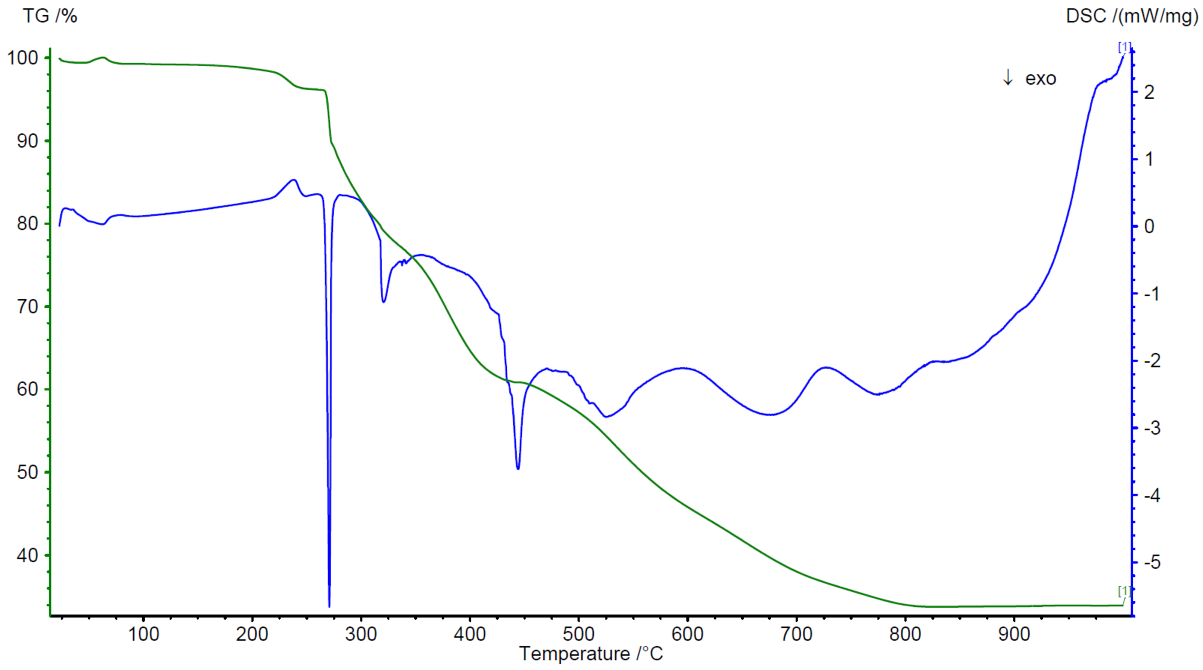

Figure S9. TG and DSC curves of **3**.

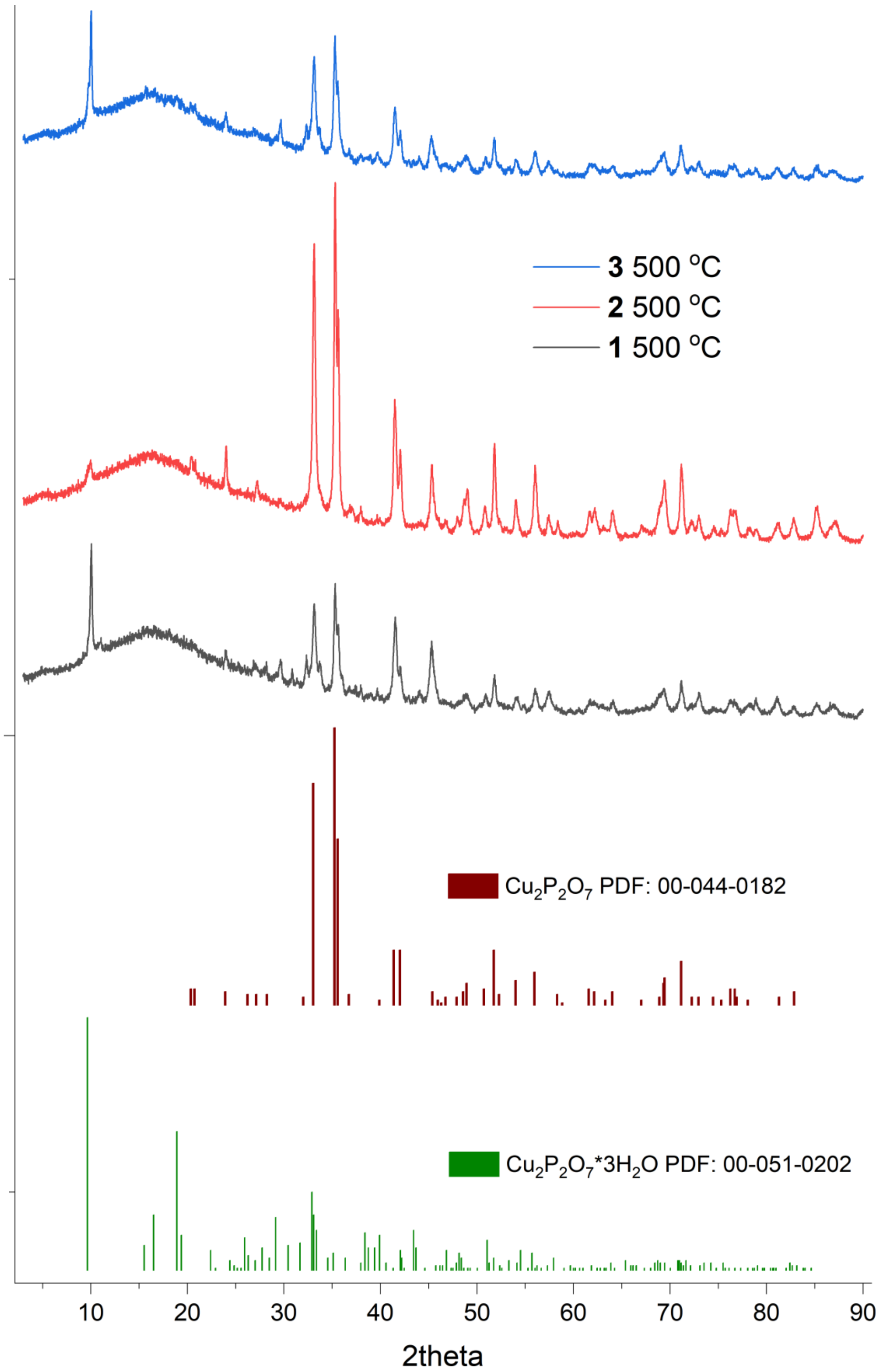

Figure S10. PXRD patterns of complexes **1**–**3** calcined at 500 °C for 10 hrs under an air atmosphere. Bar plots display PDF cards corresponding to the $Cu_2P_2O_7$ (PDF: 00-044-0182)[6,7] and $Cu_2P_2O_7 \cdot 3H_2O$ (PDF: 00-051-0202).[8]

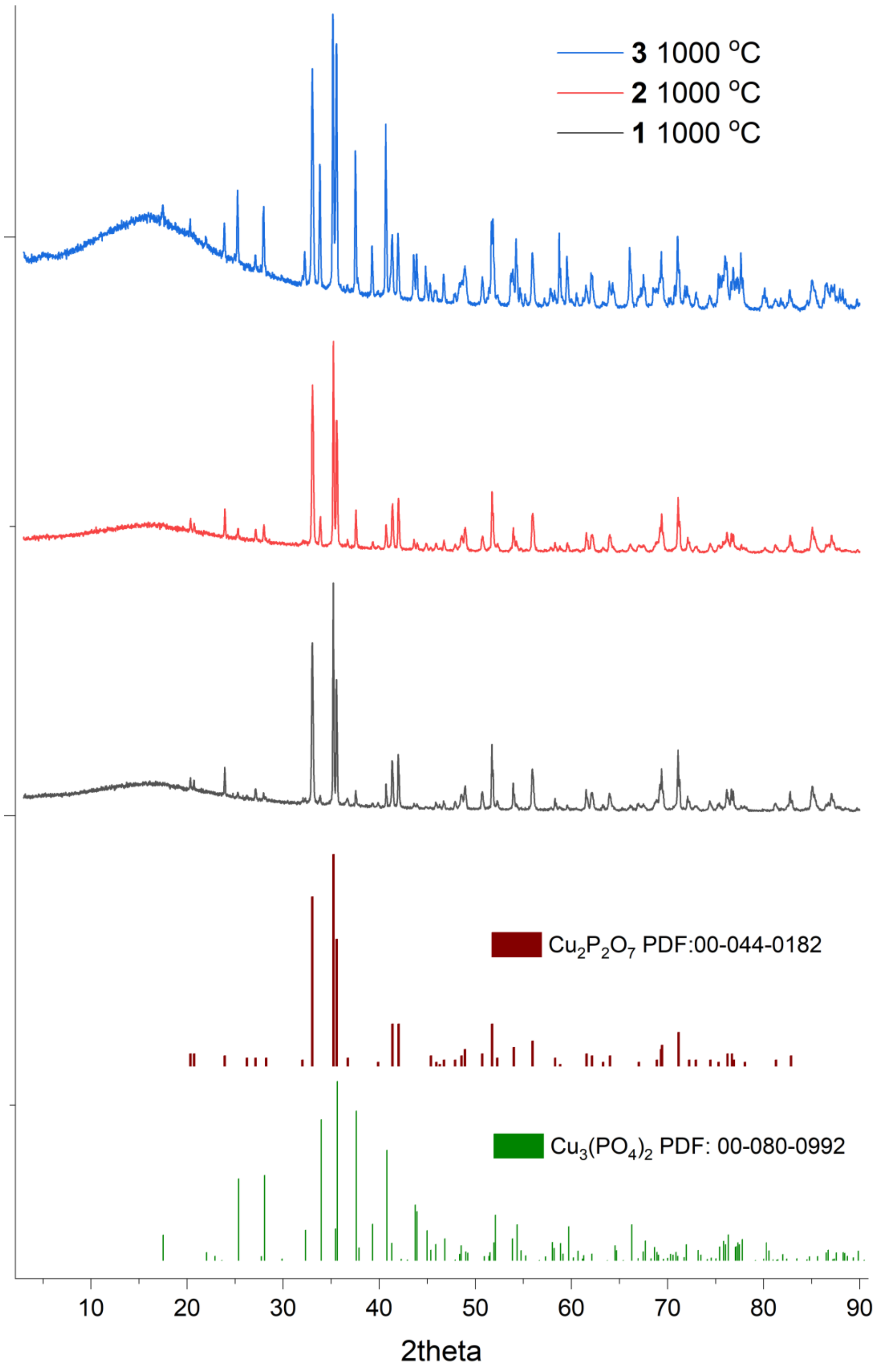

Figure S11. PXRD patterns of complexes **1**–**3** calcined at 1000 °C with the heating rate of 5 °C min^{-1} under an air atmosphere. After temperature was reached, heating was stopped. Bar plots display PDF cards corresponding to the $Cu_2P_2O_7$ (PDF: 00-044-0182)[6,7] and $Cu_3(PO_4)_2$ (PDF: 00-080-0992).[9]

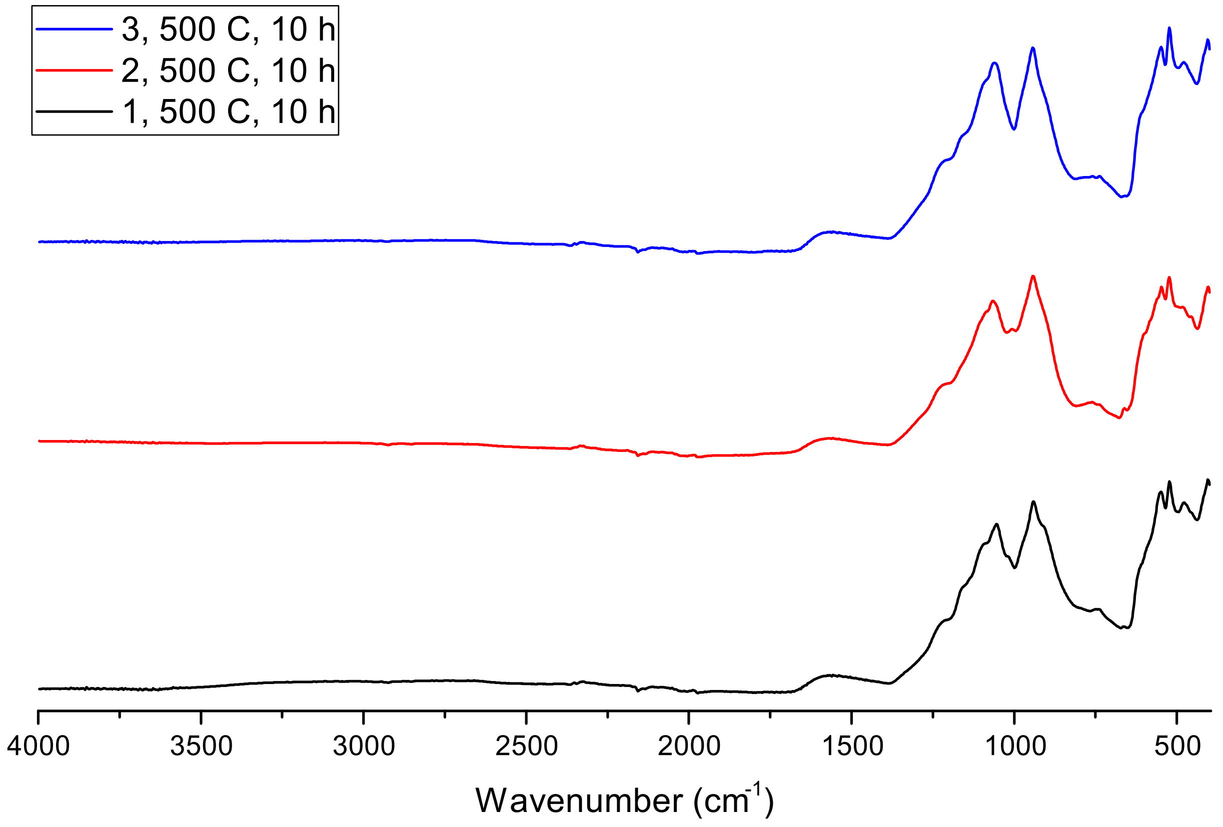

Figure S12. IR spectra of the complexes **1**–**3** calcined at 500 °C for 10 h.

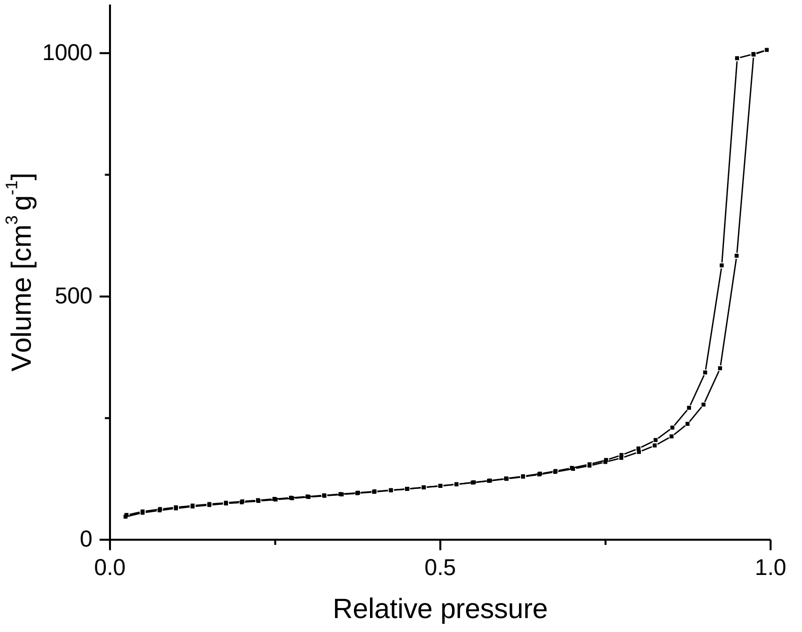

Figure S13. N_2 adsorption and desorption isotherm of Aerosil 300 used as support for the preparation of all catalysts.

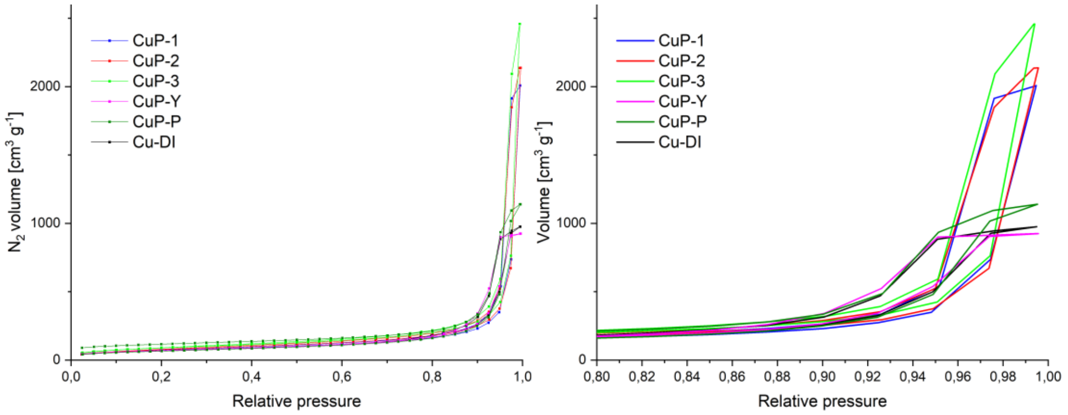

Figure S14. N_2 adsorption and desorption isotherms of the prepared catalysts and benchmark samples.

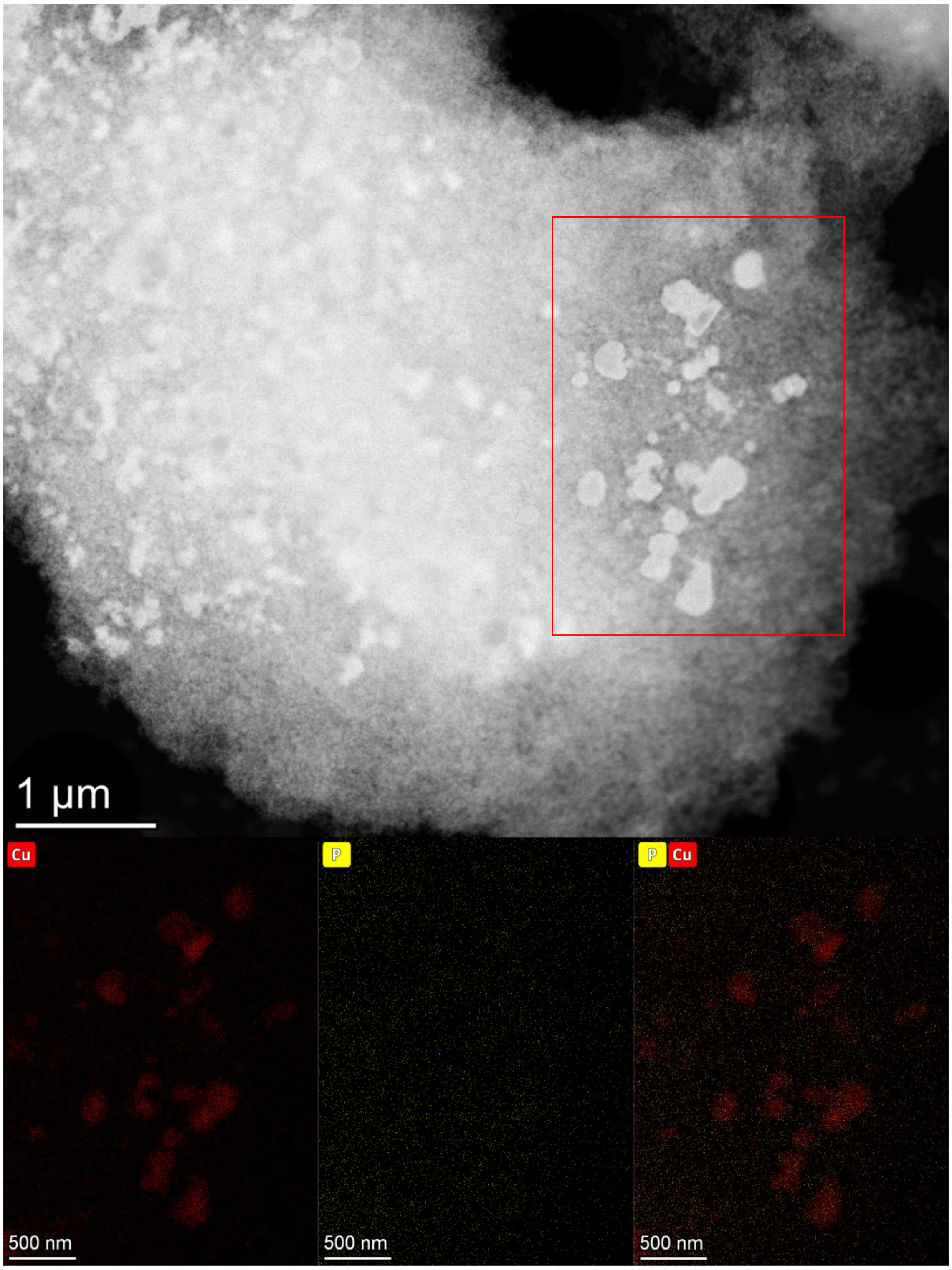

Figure S15. STEM-EDS analysis of **CuP-Y** after catalysis, red rectangle determines measured EDS area.

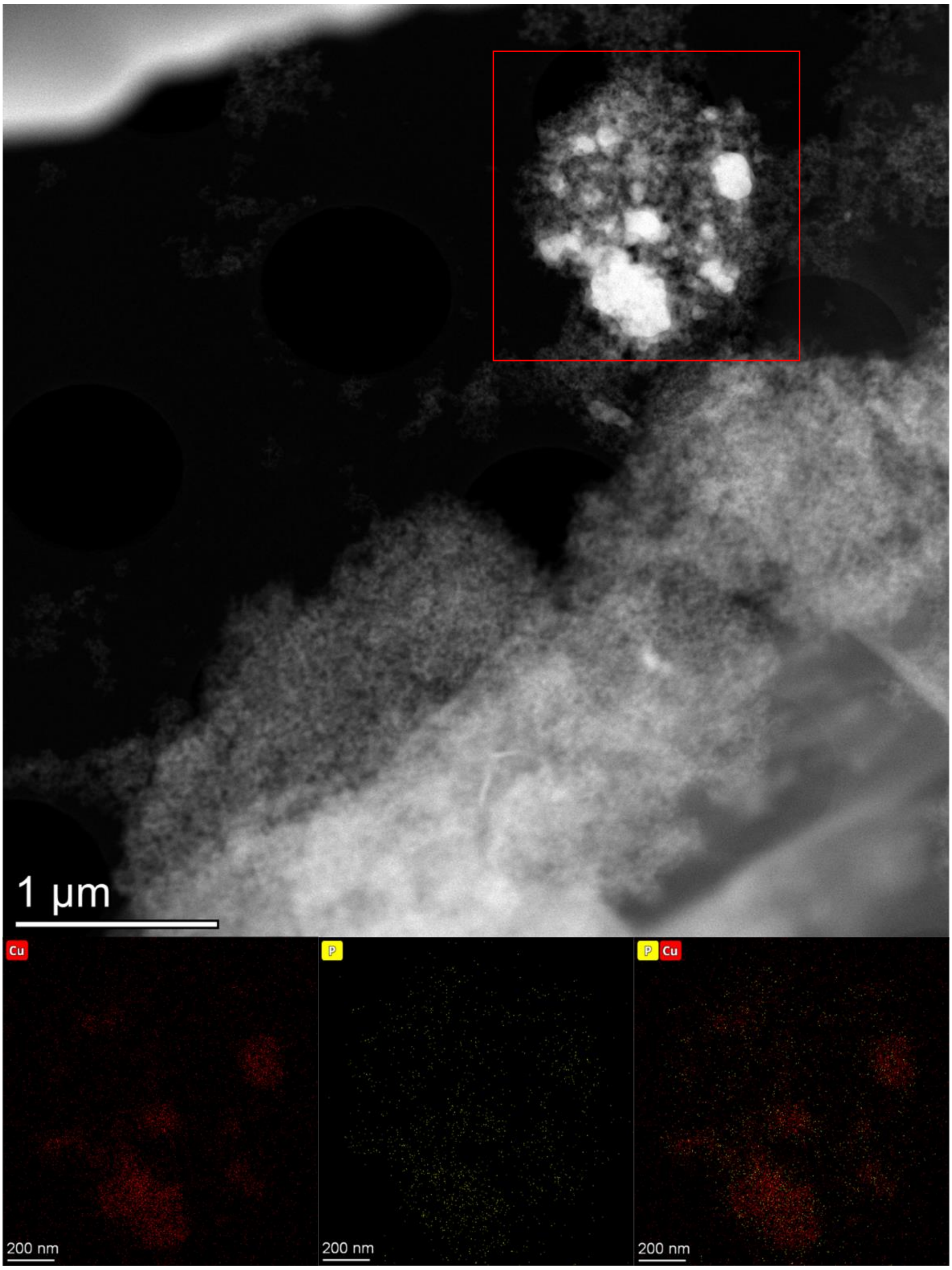

Figure S16. STEM-EDS analysis of **CuP-P** after catalysis, red rectangle determines measured EDS area.

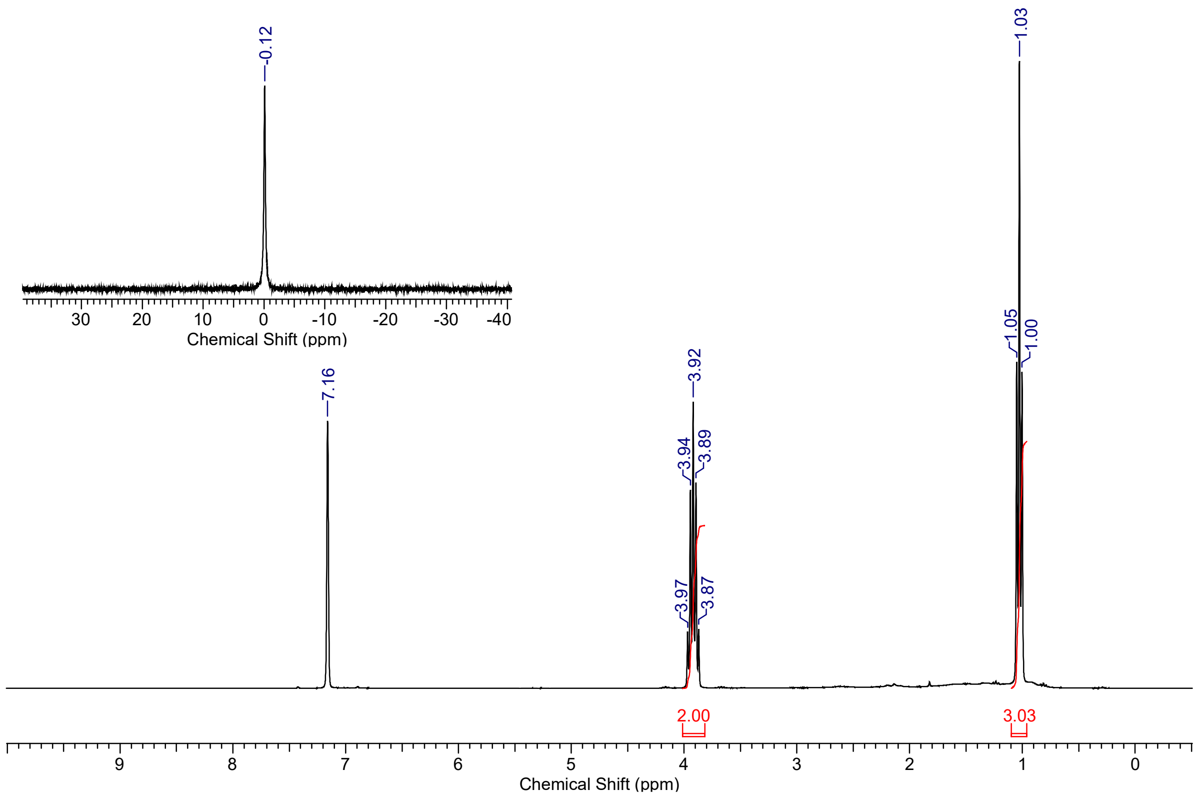

Figure S17. ^{1}H and ^{31}P {^{1}H} (inset) NMR spectra of triethyl phosphate (TEP) formed during the catalytic process on the **CuP-3-TEP** catalyst with approximately tenfold loading of Cu. Spectra were recorded in C_6D_6. The reaction products, formed during 24h time-on-stream (TOS), were collected in an ice-cooled trap. Volatile compounds were isolated using a rotary evaporator, and the residues were dissolved in 0.5 cm^3 of C_6D_6.

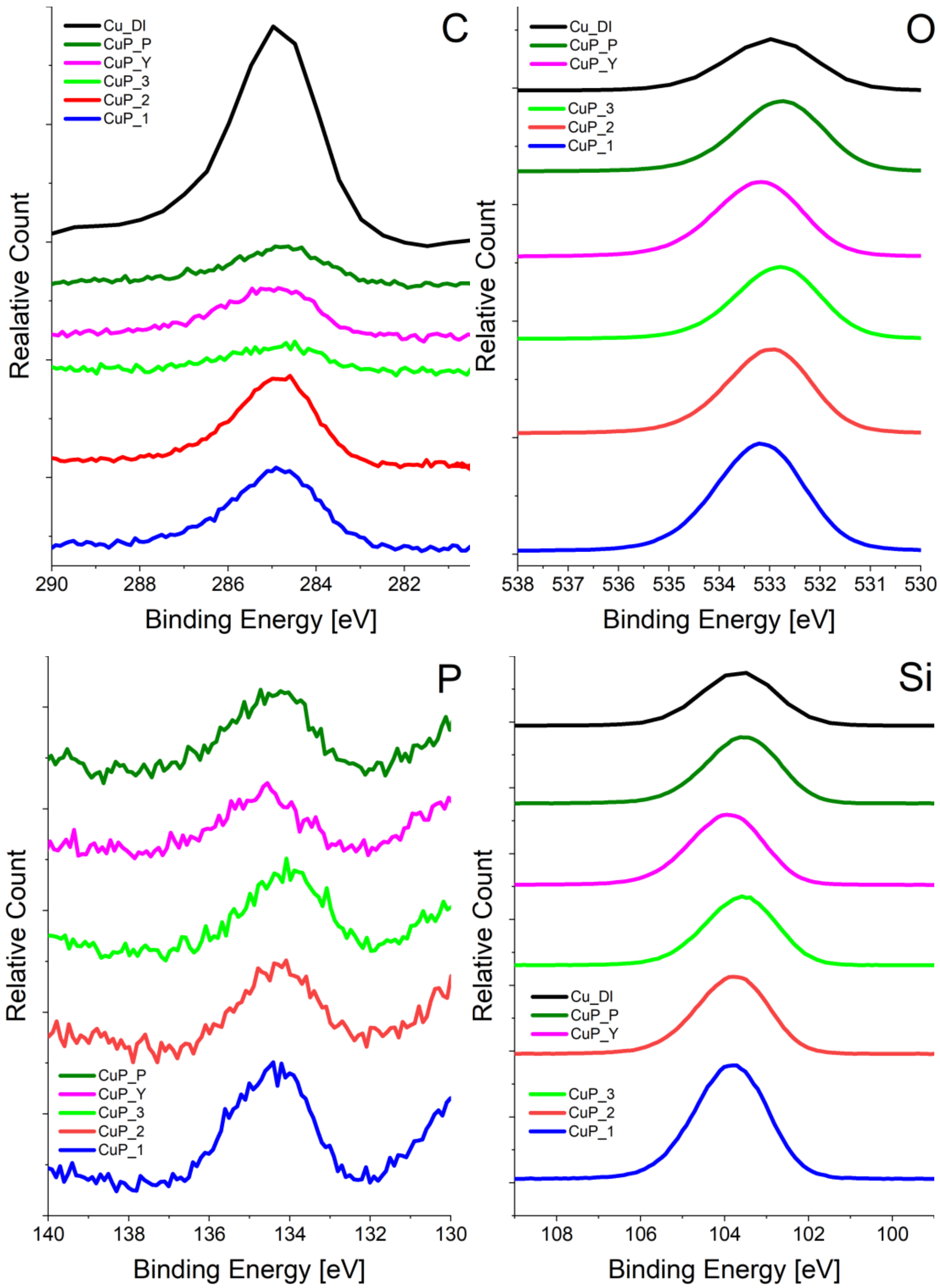

Figure S18. C 1s, O 1s, P 2p, and Si 2p XPS spectra of the freshly calcined **Cu-phosphate/SiO$_2$** samples and benchmark catalysts.

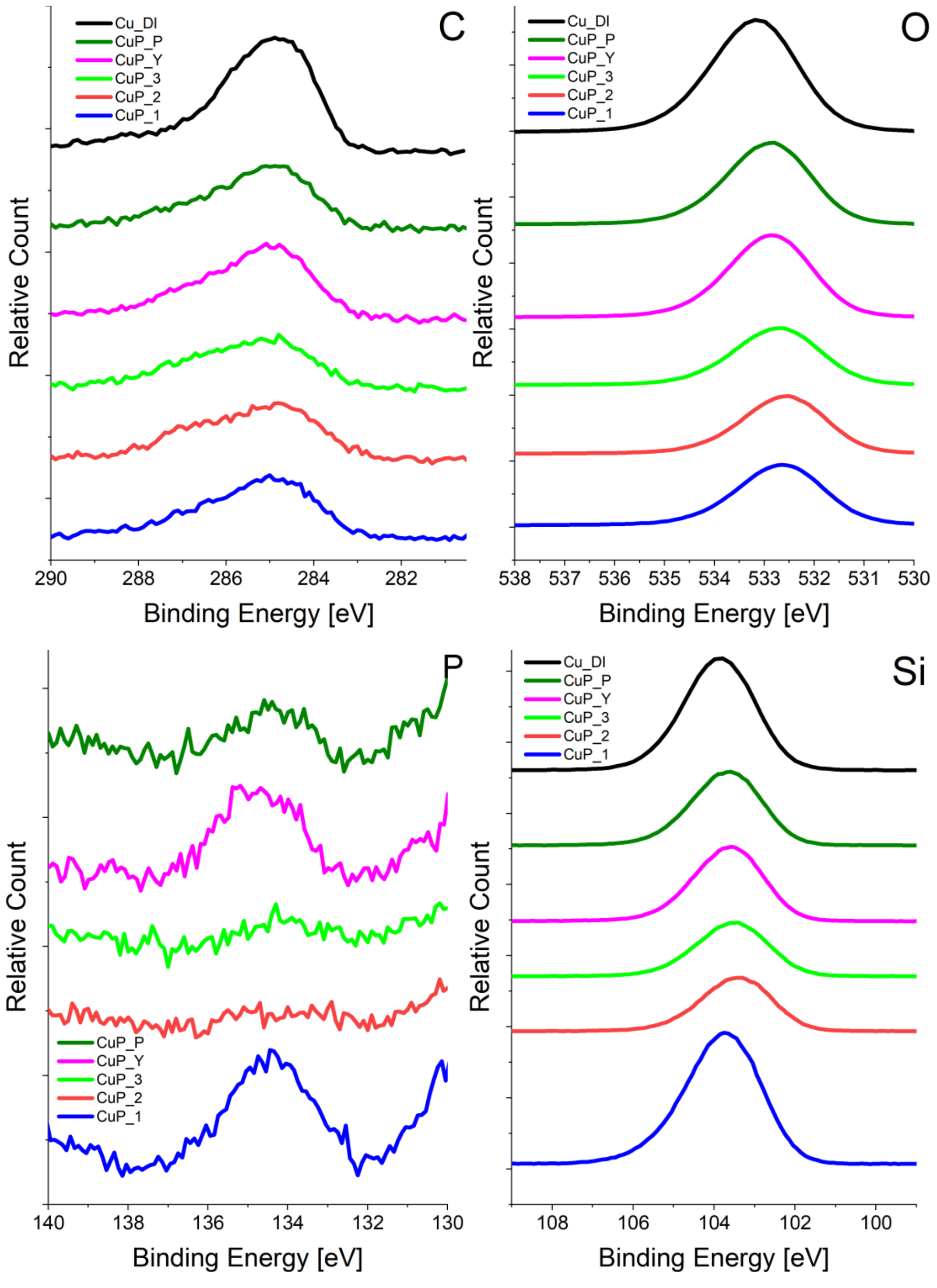

Figure S19. C 1s, O 1s, P 2p, and Si 2p XPS spectra of the spent **Cu-phosphate/SiO_2** samples and benchmark catalysts.

References

(1) Oleksyszyn, J.; Tyka, R.; Mastalerz, P. Direct Synthesis of 1-Aminoalkanephosphonic and 1-Aminoalkanephosphinic Acids from Phosphorus Trichloride or Dichlorophosphines. *Synthesis (Stuttg).* **1978**, *1978* (06), 479–480. https://doi.org/10.1055/s-1978-24792.

(2) Llunell, M.; Casanova, D.; Cirera, J.; Alemany, P.; Alvarez, S. Users Manual: SHAPE. Program for the Stereochemical Analysis of Molecular Fragments by Means of Continuous Shape Measures and Associated Tools, 2013.

(3) Pinsky, M.; Avnir, D. Continuous Symmetry Measures. 5. The Classical Polyhedra. *Inorg. Chem.* **1998**, *37* (21), 5575–5582. https://doi.org/10.1021/ic9804925.

(4) Cirera, J.; Alemany, P.; Alvarez, S. Mapping the Stereochemistry and Symmetry of Tetracoordinate Transition-Metal Complexes. *Chem. Eur. J.* **2004**, *10* (1), 190–207. https://doi.org/10.1002/chem.200305074.

(5) Alvarez, S.; Llunell, M. Continuous Symmetry Measures of Penta-Coordinate Molecules: Berry and Non-Berry Distortions of the Trigonal Bipyramid. *J. Chem. Soc. Dalt. Trans.* **2000**, No. 19, 3288–3303. https://doi.org/10.1039/b004878j.

(6) Robertson, B. E.; Calvo, C. The Crystal Structure and Phase Transformation of α-Cu2P2O7. *Acta Crystallogr.* **1967**, *22* (5), 665–672. https://doi.org/10.1107/S0365110X6700132X.

(7) Eysel, W.; Wetzel, A. ICDD Grant-in-Aid. Mineral.-Petrogr. Institut, University Heidelberg, Germany. 1992.

(8) Schneider, M.; Trommer, J.; Wilde, L.; Fratzky, D. ICDD Grant-in-Aid. Inst. f. Angewandte Chemie, Berlin, Germany. 1999. https://www.icdd.com/grant-in-aid/.

(9) Forsyth, J. B.; Wilkinson, C.; Paster, S.; Effenberger, H. The Antiferromagnetic Structure of Triclinic Copper(II) Phosphate. *J. Phys. Condens. Matter* **1990**, *2* (6), 1609–1617. https://doi.org/10.1088/0953-8984/2/6/019.
